# Dual-source DPP4 drives intestinal fibrosis in Crohn’s disease: synergistic therapeutic targeting of host and microbiota pathways

**DOI:** 10.1080/19490976.2025.2593119

**Published:** 2025-12-03

**Authors:** Jiajia Li, Ying Xu, Mingyuan Wang, Junjie Lin, Junjian Sun, Jingjing Ma, Hongjie Zhang

**Affiliations:** a Department of Gastroenterology, The First Affiliated Hospital with Nanjing Medical University, Nanjing, Jiangsu Province, People’s Republic of China; b Department of Gastroenterology, Nanjing First Hospital, Nanjing Medical University, Nanjing, Jiangsu Province, People’s Republic of China

**Keywords:** Crohn's disease, intestinal fibrosis, membrane-bound DPP4, soluble DPP4, gut microbiota-derived DPP4, human intestinal myofibroblasts, metagenomic sequencing, engineered bacteria

## Abstract

Crohn’s disease (CD), a chronic inflammatory bowel disorder, often progresses to intestinal fibrosis and stricture, yet no effective anti-fibrotic treatments exist. This study reveals dipeptidyl peptidase 4 (DPP4) as a pivotal driver of fibrosis through bioinformatics analysis, clinical samples, and experimental models. Elevated DPP4 expression was observed in stenotic intestinal tissues of CD patients and dextran sodium sulfate (DSS)-induced fibrotic mice. Mechanistically, both membrane-bound DPP4 and soluble DPP4 (sDPP4) activated human intestinal myofibroblasts (HIMFs) via the PI3K-AKT pathway, stimulating migration, proliferation, and extracellular matrix deposition. Importantly, metagenomic sequencing revealed enrichment of microbial *Dpp4* genes in fecal samples from CD patients with stenosis, and *in vivo* colonization with engineered *E. coli* overexpressing microbial DPP4 exacerbated fibrotic remodeling, confirming microbiota-derived DPP4 (mDPP4) as a pathogenic driver. Furthermore, pharmacological inhibition of host DPP4 (sitagliptin) or selective blockade of mDPP4 (Dau-d4) attenuated fibrosis in murine models, with combined therapy showing enhanced efficacy. These findings underscore the roles of DPP4, originating from both host and microbiota, and existing in membrane-bound and soluble forms, in promoting CD-associated intestinal fibrosis. This study identifies DPP4 as a novel therapeutic target, proposing dual-source inhibition as a promising strategy to prevent stricture formation in CD patients, thereby addressing a critical unmet clinical need.

## Graphical abstract

Host- and microbiota-derived DPP4 synergistically drive intestinal fibrosis in Crohn’s disease (CD). Membrane-bound DPP4 and soluble DPP4 (sDPP4) activate human intestinal myofibroblasts (HIMFs) via the PI3K-AKT pathway, promoting migration and extracellular matrix deposition. Microbiota-derived DPP4 (mDPP4) further amplifies fibrosis. Inhibition of host (sitagliptin) and microbial (Dau-d4) DPP4 mitigates fibrosis, with dual targeting showing enhanced efficacy. These findings identify DPP4 as a potential therapeutic target for CD-associated strictures.

**Figure:**
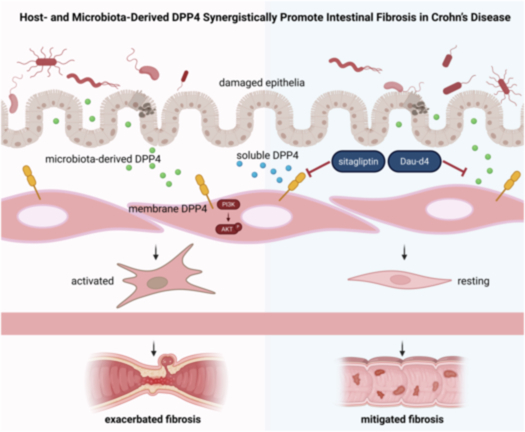

## Introduction

Crohn’s disease (CD) is a chronic inflammatory bowel disorder characterised by relapsing-remitting symptoms. Approximately 50% of patients develop intestinal fibrosis and strictures, leading to abdominal pain, abdominal distension, and vomiting.^1^ The primary management for CD with fibrotic stenosis involves endoscopic and surgical removal of the affected segments; however, these procedures often require repeated interventions and may substantially affect patients’ quality of life.^2^ Nevertheless, there remains a critical lack of therapies that directly target fibrotic progression, underscoring the need for novel anti-fibrotic strategies.

Fibrosis is pathologically characterised by abnormal deposition of extracellular matrix (ECM) and dynamic remodelling of cellular composition.^3^ Myofibroblasts, identified by their expression of *α*-smooth muscle actin (*α*-SMA), serve as central mediators of fibrogenesis.^4^ These cells originate from various precursor cells, with fibroblasts identified as the primary source.^5^ Recent studies have highlighted the critical role of myofibroblast activation and soluble mediators in the development of intestinal fibrosis in CD, but the exact underlying mechanisms are still not fully understood.^3^

With advances in microbiome research and metabolomics, the role of gut microbiota in the pathogenesis and progression of CD has become increasingly evident. The gut microbiota could influence the onset and progression of intestinal inflammation through a variety of substances, including lipopolysaccharides (LPS), metabolites such as short-chain fatty acids (SCFAs) and secondary bile acids, bacterial proteins like peptidoglycan and other proteins, along with bacterial toxins. These bioactive substances exert their effects by altering epithelial barrier function and modulating the immune system, both directly and indirectly. Notably, recent research has implicated the involvement of gut microbiota in the formation of intestinal strictures in CD. Xu et al. demonstrated that CD-associated adherent-invasive Escherichia coli (AIEC) can suppress the expression of let-7b in intestinal epithelial cell-derived exosomes, leading to altered macrophage polarisation and enhanced intestinal fibrosis.^6^ These findings underscore the need for further investigation into the precise role and molecular mechanisms by which gut microbiota contributes to CD-related fibrosis.

Dipeptidyl peptidase 4 (DPP4, also known as CD26) is a serine protease that plays a crucial role in glucose metabolism. It exerts its enzymatic activity by cleaving *N*-terminal dipeptides from various substrates, including glucagon-like peptide−1 (GLP−1), neuropeptides, and chemokines. Through its enzymatic function, DPP4 regulates insulin secretion and serves as a key therapeutic target in type 2 diabetes mellitus, where its inhibition enhances GLP−1 activity to promote glycemic control.^7^ Beyond its role in metabolism, DPP4 has been implicated in immune modulation and fibrosis. It exists in both a membrane-bound form and a soluble form (sDPP4), which is generated through proteolytic cleavage.^8^ While DPP4’s involvement in renal and skin fibrosis is well-documented,^9‐11^and sDPP4 has been proposed as a biomarker for IBD activity,^12^ its functional contribution to intestinal fibrosis in CD remains poorly understood.

Herein, we found that both membrane-bound and soluble DPP4 levels were elevated in CD patients with stenosis compared to those without stenosis. Strikingly, faecal levels of gut microbiota-derived DPP4 were also significantly increased in CD patients with stenosis, suggesting a synergistic interplay between host- and microbiota-derived DPP4 in driving fibrotic progression. We therefore aimed to elucidate the roles of DPP4 from distinct sources (host and gut microbiota) and in different forms (membrane-bound and soluble) in CD-associated intestinal fibrosis, and to identify synergistic therapeutic strategies targeting both pathways.

## Materials and methods

### Bioinformatics analysis

The GSE66207 dataset was retrieved from the Gene Expression Omnibus (GEO) database. Bulk RNA sequencing (RNA-seq) data were analysed using the *limma* package in R (version 4.0.1) to identify differentially expressed genes (DEGs) between B2 (stricturing) and B1 (non-stricturing) CD samples, with significance thresholds set at |log2 fold change| >1 and *p*-value <0.05. Weighted Gene Co-expression Network Analysis (WGCNA) was applied to detect co-expression modules among these DEGs. Protein-protein interaction (PPI) networks were constructed using the STRING database and visualised in Cytoscape. Hub genes were predicted using the CytoHubba plugin. Similarly, RNA-seq data from intestinal fibroblasts isolated from paired normal and stenotic CD tissues (GSE90607) were analysed to compare DPP4 expression.

To further investigate the signalling pathways associated with DPP4 in intestinal fibrosis, we re-analysed a single-cell RNA sequencing dataset of full-thickness intestinal tissues from stricturing CD patients, which was kindly provided by Dr. Florian Rieder (Lerner Research Institute, Cleveland Clinic).^13^ The fibroblast compartment used for our analysis was derived from stenotic segments of CD resections. Fibroblasts were extracted from the dataset and stratified into DPP4⁺ and DPP4⁻ subpopulations based on DPP4 expression levels. Differential gene expression analysis between these two subpopulations was performed using the Seurat package (version 5.1.0) in R, followed by KEGG pathway enrichment analysis of DEGs using the clusterProfiler package (version 4.14.4) to identify signalling pathways preferentially enriched in DPP4⁺ fibroblasts.

### Human samples

CD patients with or without intestinal stenosis and age- and sex-matched control subjects were enroled at the First Affiliated Hospital of Nanjing Medical University between June 2022 and September 2023. The diagnosis of CD was established according to standardised clinical, endoscopic, radiologic, and histopathologic criteria.^2^ The definition of fibrostenotic CD was based on the international consensus proposed by Rieder et al.^1^ Specifically, fibrotic stenosis was defined by the presence of the following features: (1) Endoscopic evidence of luminal narrowing that prevents the passage of an adult colonoscope without prior endoscopic dilation with a reasonable amount of pressure applied; (2) Cross-sectional imaging (CT or MR enterography) demonstrating (a) a luminal diameter reduction of ≥50% compared with the adjacent normal bowel, (b) bowel wall thickening of ≥25% relative to non-affected segments, and (c) pre- stricture dilation with a luminal diameter >3 cm; (3) Histopathologic confirmation of transmural fibrosis by Masson’s trichrome staining of resected tissue. Exclusion criteria included: (1) Age <18 years; (2) Pregnancy or lactation; (3) Current use of DPP4 inhibitors; (4) Concurrent autoimmune diseases (e.g., rheumatoid arthritis, systemic lupus erythematosus). The control group comprised individuals undergoing intestinal resection for non-inflammatory conditions (small intestinal diverticulum or stromal tumours), with no evidence of active inflammation or fibrosis on histology. Demographic variables (age, sex) were matched between CD and control groups. To enable accurate assessment of DPP4 expression, CD patients were stratified into stenotic and non-stenotic subgroups for blood and faecal sample analysis. These subgroups were matched for Crohn’s Disease Activity Index (CDAI) to minimise confounding by inflammation severity. Surgically resected intestinal tissues (stenotic/non-stenotic regions in CD patients; normal tissues in controls), peripheral blood (3 mL), and faecal samples (500 mg) were collected for downstream analysis. Clinical characteristics of the enroled subjects are summarised in Supplementary Table 1.

### Isolation of primary human intestinal myofibroblasts (HIMFs)

Primary HIMFs were isolated from surgically resected intestinal tissues of CD patients (with/without stenosis) and non-inflammatory controls, following a modified explant culture protocol as previously described by Rieder et al.^13^^,^^14^ Briefly, mucosal layers were aseptically dissected from full-thickness intestinal specimens, and the lamina propria was mechanically separated. Tissue segments (2–3 cm) were incubated with 1 mM dithiothreitol (DTT; Sigma-Aldrich, USA) in Hank’s Balanced Salt Solution (HBSS; Corning, USA) for 30 minutes to remove residual mucus, followed by three washes in HBSS containing 2.5% penicillin-streptomycin (Biosharp, China) to minimise microbial contamination. The lamina propria strips were then minced into 1 × 1 mm fragments using sterile scalpels and evenly distributed onto pre-scored 100 mm culture dishes (Corning, USA), with a small amount of Dulbecco’s Modified Eagle Medium (DMEM, Gibco, USA) supplemented with 10% heat-inactivated foetal bovine serum (FBS, Gibco, USA) and 1% penicillin-streptomycin. Culture medium was replaced 24 hours after plating to remove non-adherent debris and then refreshed twice per week. Passage 1 cells were harvested for RNA and protein extraction to analyse differential gene expression between stenotic and non-stenotic samples, whereas cells from passages 3 to 10 were utilised for subsequent functional experiments.

### Cell culture

Primary HIMFs were cultured at 37 °C in a humidified 5% CO_2_ incubator and grown in DMEM supplemented with 10% FBS and 1% penicillin-streptomycin. To establish a fibroblast activation model, primary HIMFs were stimulated with recombinant human TGF-*β* (Novoprotein, China) at a concentration of 10 ng/mL for 48 hours.

### Multiplex immunohistochemistry (mIHC)

Multiplex immunohistochemistry was performed on formalin-fixed, paraffin-embedded (FFPE) colonic tissues from healthy controls, non-stenotic CD patients, and stenotic CD patients using the PANO Multiplex IHC kit (Panovue, China) according to the manufacturer’s protocol. Briefly, sections were deparaffinized, rehydrated, and subjected to microwave-assisted antigen retrieval. After cooling, slides were blocked and sequentially incubated with primary antibodies, HRP-conjugated secondary antibodies, and TSA-conjugated fluorophores. Each target antigen was detected through multiple rounds of antigen retrieval, blocking, and staining. DAPI was used for nuclear counterstaining. The primary antibodies used included anti-DPP4 (Abcam, UK), anti-CD3 (Abcam, UK), anti-CD90 (Abcam, UK), and anti-EpCAM (CST, USA). Slides were scanned using the PanoScanner 20 (Panovue, China).

### Enzyme-linked immunosorbent assay (ELISA)

The concentration of DPP4 in human serum was measured using a human DPPIV/CD26 ELISA kit (MultiSciences, China) according to the manufacturer’s instructions. For serum samples, 80 μL of 1 × assay buffer and 20 μL of pre-diluted sample were added to the sample wells. Next, 50 μL of diluted detection antibody (1:100) was added to each well, and the plate was sealed and incubated at 100−300 rpm for 2 hours at room temperature. After washing the plate 6 times with 300 μL of wash buffer per well, 100 μL of diluted HRP-labelled streptavidin (1:100) was added, and the plate was incubated again at 100−300 rpm for 45 minutes. TMB substrate (100 μL) was then added, and the plate was incubated in the dark for 5−30 minutes at room temperature. The reaction was stopped with 100 μL of stop solution. Absorbance was measured at 450 nm and 570 nm, and corrected optical density (OD) values were calculated by subtracting the 570 nm readings from the 450 nm readings. Finally, the concentration of DPP4 in the serum was calculated using a standard curve.

### Histological assessment

Colonic tissues were fixed in 4% paraformaldehyde for 48 hours, paraffin-embedded, and sectioned at 4 μm thickness. For hematoxylin and eosin (H&E) staining, sections were dewaxed in xylene, rehydrated through graded ethanol series, and stained using standard protocols. The inflammation score of chronic colitis models was determined by H&E staining as previously reported.^15‐17^ For immunohistochemistry (IHC), antigen retrieval was performed by boiling sections in 10 mM sodium citrate buffer (pH 6.0) for 20 minutes. After blocking, sections were incubated overnight at 4 °C with anti-DPP4 antibody (1:100, Abmart, China). A biotinylated secondary antibody and HRP-conjugated polymer were sequentially applied at 37 °C for 30 minutes each. Collagen deposition was quantified on Masson’s trichrome-stained sections using ImageJ (NIH, USA) with colour deconvolution plugin. The collagen volume fraction (%) was calculated as the ratio of collagen-positive area (blue) to total tissue area across five random fields per sample.

### Immunofluorescence

Immunostaining of tissue sections was performed after deparaffinization and antigen retrieval in citrate buffer. Nonspecific binding was blocked by incubation with 10% normal goat serum (Biosharp, China) for 1 hour at room temperature. The slides were then incubated overnight at 4 °C with primary antibodies: DPP4 (1:100, Abmart, China) and *α*-SMA (1:100, Santa Cruz, USA). After washing, secondary antibodies (goat anti-rabbit IgG Alexa Fluor 488 and goat anti-mouse IgG Alexa Fluor 594; 1:400, Jackson ImmunoResearch, USA) were applied. Nuclei were stained with DAPI (Biosharp, China). Immunostained slides were photographed using a Leica THUNDER Imager (Leica Microsystems, Germany) under consistent exposure settings. The thickness of the muscularis propria was quantified using ImageJ.^18^

### Dextran sodium sulphate (DSS)–induced chronic colitis model

Male C57BL/6 mice (8 weeks old) were obtained from Animal Core Facility of Nanjing Medical University. Chronic colitis was induced through three cycles of DSS administration. Each cycle consisted of 7 days of 1.5% dextran sodium sulphate (DSS, MP Biomedicals, USA) in drinking water followed by a 14-day recovery period with tap water. During the third cycle (days 43−63), mice were treated with sitagliptin (10 mg/kg; MCE, USA), either alone or in combination with the microbial DPP4 inhibitor Dau-d4 (10 mg/kg; MCE, USA). Both treatments were administered via oral gavage three times per week throughout the third cycle. Mice were euthanized on day 77 (14 days after completing the third cycle) for downstream analysis. Colon tissues were harvested for subsequent histological scoring and immunofluorescence.

### Construction of engineered *E. coli* overexpressing microbial DPP4 and *in vivo* functional validation

An engineered *E. coli* MG1655 strain stably overexpressing btDPP4 was constructed by Forhigh Biotech (Hangzhou, China) using CRISPR-Cas9-mediated genome integration. Briefly, the btDPP4 coding sequence (signal peptide removed) was fused in-frame with the pelB leader sequence and inserted into the attλ locus of the MG1655 genome under the control of the tac promoter. For homologous recombination, the btDPP4 fragment flanked by attλ upstream/downstream arms was assembled into the attλ-pUXT vector by seamless cloning and confirmed by colony PCR and Sanger sequencing. The repair template and attλ sgRNA plasmid were co-electroporated into MG1655 cells pre-harbouring a Cas9 expression plasmid. Recombinants were selected on kanamycin/spectinomycin plates, and positive clones were verified by colony PCR using attλ-specific primers, yielding the expected 2.6 kb insertion product. The Cas9 and sgRNA plasmids were subsequently cured, and the final engineered strain (*E. coli* btDPP4) was confirmed by sequencing.

To evaluate the *in vivo* role of microbial DPP4, the engineered strain was introduced into the DSS-induced chronic colitis model. Prior to bacterial gavage, the gut microbiota of mice was depleted by administering an antibiotic cocktail (0.2 g/L ampicillin, neomycin, and metronidazole, and 0.1 g/L vancomycin) in drinking water for 3 consecutive days. After antibiotic pretreatment, mice were randomly assigned to three groups: (1) DSS + PBS (daily gavage with sterile PBS), (2) DSS + *E. coli* WT (1 × 10⁸ CFU of wild type *E. coli* MG1655), and (3) DSS + *E. coli* btDPP4 (1 × 10⁸ CFU of *E. coli* btDPP4), with bacterial or PBS gavage administered once daily throughout the DSS modelling period. At the end of the third recovery cycle, mice were sacrificed for downstream analysis.

### Metagenomic sequencing and functional annotation

Metagenomic sequencing was performed following standard protocols with minor modifications. Briefly, faecal samples were collected from controls (*n* = 10), CD patients without intestinal stenosis (*n* = 20), and CD patients with confirmed intestinal stenosis (*n* = 20). None of the enroled participants had received systemic or oral antibiotic treatment within three months before sample collection. Total DNA was extracted using the QIAamp PowerFecal Pro DNA Kit following the manufacturer’s instructions. DNA quality and quantity were assessed by NanoDrop spectrophotometry and agarose gel electrophoresis. Sequencing libraries were prepared using the Ultra DNA Library Prep Kit for Illumina (NEB, USA) following the manufacturer’s instructions. Genomic DNA was fragmented, end‑repaired, A‑tailed, and ligated to Illumina sequencing adaptors, followed by PCR amplification and purification using AMPure XP beads (Beckman Coulter, USA). Library quality and insert size were assessed with a Qubit 2.0 fluorometer (Thermo Fisher Scientific, USA) and an Agilent 2100 Bioanalyzer (Agilent Technologies, USA). Quantified libraries (>3 nM) were subjected to cluster generation on a cBot system and sequenced on the Illumina HiSeq 4000 platform to generate 150‑bp paired‑end reads.

Raw reads were quality‑controlled by trimming adapters and low‑quality bases (PHRED score <30) using fastp. Host‑derived sequences were removed by aligning reads to the human genome (hg38) with minimap2. High‑quality non‑host reads were then profiled for taxonomic composition using MetaPhlAn4 (v4.0) with the mpa_vJan21_CHOCOPhlAn database under default parameters. Functional profiling was performed using HUMAnN3, which mapped reads to UniRef90 and KEGG databases to quantify pathway and gene family abundances. Specifically, DPP4 homologues were identified based on KEGG ortholog K01278 (dipeptidyl peptidase IV). For visualisation of taxonomic contributions, DPP4-encoding reads were summarised at both the genus and species levels. Representative taxa were extracted and plotted to highlight dominant contributors.

### Determination of DPP4 activity

DPP4 activity in human and murine faecal samples was quantified using Gly-Pro-*p*-nitroanilide hydrochloride (Gly-Pro-pNA, TargetMol, USA) as the substrate. Briefly, 60 mg of fresh stool was homogenised in RIPA lysis buffer supplemented with protease inhibitors using bead-beating and sonication to ensure thorough cell lysis. After centrifugation (12,000 × g, 10 min, 4 °C) to remove particulate debris, the supernatant was incubated with Gly-Pro-pNA substrate at 37 °C, and DPP4 activity was quantified by continuously monitoring the release of *p*-nitroaniline (pNA) at 405 nm. Enzyme activity was normalised to faecal wet weight [expressed as nmol/(min**·**mg faeces)].

For the assessment of secreted microbial DPP4 activity, *Bacteroides thetaiotaomicron* (ATCC 29148) was cultured anaerobically in Brain Heart Infusion (BHI) medium at 37 °C. Bacterial growth was monitored by measuring OD600 at indicated time points. To evaluate secreted DPP4 activity, culture supernatants were harvested at 8 h, 16 h, and 24 h by centrifugation at 12,000 × g for 20 min at 4 °C, followed by filtration through 0.22 μm filters to remove residual bacterial cells. The supernatants were then concentrated 10-fold using a 10 kDa molecular weight ultrafiltration device (Millipore, USA) to enrich for extracellular DPP4. DPP4 enzymatic activity in the concentrated supernatants was assessed as mentioned above and was calculated as nmol of pNA released per min per mL of supernatant. Culture medium subjected to identical processing was used as a negative control.

### Recombinant expression and purification of microbial DPP4 protein

The codon-optimised bt*Dpp4* gene (*Bacteroides thetaiotaomicron* DPP4 homologue) was synthesised (Tsingke Biotech) and cloned into the pET28a vector using NcoI/XhoI restriction sites, generating the recombinant plasmid pET28a-BtDPP4. Following sequencing and restriction digestion verification, the plasmid was transformed into *E. coli* BL21(DE3) competent cells. Bacterial cultures were grown in LB medium containing kanamycin (50 µg/mL) at 37 °C until OD600 reached 0.5–0.6, and protein expression was induced with 0.2 mM IPTG for 15–18 h. Cells were harvested, lysed by sonication, and centrifuged (12,000 × g, 30 min, 4 °C). The supernatant was purified via Ni-NTA affinity chromatography using TBS buffer (20 mM Tris-HCl, 0.5 M NaCl, pH 8.0) with stepwise imidazole elution (20–250 mM). Purified protein was concentrated and analysed by SDS-PAGE, confirming purity greater than 85%. The amino acid sequence of btDPP4 is provided in Supplementary Table 2. Purified btDPP4 was aliquoted and stored at −80 °C until use.

### Flow cytometry of surface DPP4

Primary HIMFs were harvested, washed with PBS, and incubated in the dark with anti-human DPP4-PE antibody (BioLegend, USA) or isotype control for 30 min at 4 °C. Cells were washed with PBS and subsequently analysed using a BD FACS flow cytometer and FlowJo software.

### Ki67 immunofluorescence staining

For the immunostaining of HIMFs, the cells were fixed in 4% paraformaldehyde and permeabilized with 0.1% Triton X−100 (Biosharp, China). After blocking with 10% normal goat serum for 1 hour, cells were incubated overnight at 4 °C with an anti-Ki67 antibody (1:1000, Abcam, USA). After three PBS washes, Alexa Fluor 594-conjugated goat anti-rabbit IgG was applied for 1 hour at room temperature. Subsequent procedures, including nuclear staining with DAPI, were consistent with standard immunofluorescence protocols. The percentage of Ki67-positive cells was quantified using ImageJ.

### Scratch wound healing assay

Primary HIMFs were seeded in 6-well plates and allowed to adhere for 24 hours. The culture medium was then replaced with serum-free medium, and the cells were incubated overnight under serum starvation conditions. Confluent HIMF monolayers were scratched using a 200 µL pipette tip to create uniform wounds. Following wounding, the cells were treated with TGF-*β* (10 ng/mL, Novoprotein, China), the DPP4 inhibitor sitagliptin (20 nmol/L, MCE, USA), sDPP4 (200 ng/mL, MCE, USA), mDPP4 (200 ng/mL), or the mDPP4 inhibitor Dau-d4 (100 nmol/L, MCE, USA). Wound images were captured at 0 hours and 24 hours post-treatment using an inverted microscope. The migration area percentage was quantified using ImageJ software by comparing the wound area at 0 hours and 24 hours.

### Western blot (WB) analysis

Cells or tissues were lysed in RIPA lysis buffer (Beyotime, China) containing 1% protease/phosphatase inhibitor cocktail (Beyotime, China). Protein concentrations were determined using a BCA assay (Beyotime, China), and typically 20–40 μg of protein per lane was separated by electrophoresis on 10% SDS-PAGE gels. The separated proteins were then transferred to PVDF membranes, followed by blocking with 5% skim milk to reduce nonspecific binding. Primary antibodies (1:1000) including those targeting ERK, *p*-ERK, JNK, *p*-JNK, AKT, *p*-AKT, p38, *p*-p38, PI3K, and *p*-PI3K (CST, USA), as well as antibodies targeting DPP4 (Abmart, China) and *α*-SMA (SANTACRUZ, USA), were incubated with the membranes overnight at 4 °C. This was followed by incubation with HRP-conjugated secondary antibodies (1:5000, Proteintech, China) for 1 hour at room temperature. Protein signals were detected using an enhanced chemiluminescence (ECL) kit (Millipore, USA). Data were analysed with ImageJ software and normalised to GAPDH protein expression.

For bacterial protein analysis, cultures of MG1655 wild-type (*E. coli* WT) and MG1655 strains overexpressing His-tagged btDPP4 (*E. coli* btDPP4) were harvested and centrifuged at 4,000 rpm for 15 min at 4 °C. The bacterial pellets were resuspended in RIPA lysis buffer and lysed by sonication, followed by centrifugation to remove debris. Equal amounts of bacterial protein were separated on 10% SDS-PAGE gels and transferred to PVDF membranes. After blocking with 5% skim milk, membranes were incubated with an anti-His tag primary antibody (Proteintech, USA) overnight at 4 °C, followed by HRP-conjugated secondary antibody for 1 hour at room temperature. Protein signals were visualised using ECL as described above.

### RNA extraction and quantitative real-time PCR (RT-qPCR)

Total RNA was extracted from cellular and tissue samples using Trizol reagent (Takara, Japan) and reverse-transcribed into cDNA using a reverse transcription kit (Vazyme, China). For the detection of microbial DPP4, total RNA was extracted from faecal samples using the RNeasy PowerFecal Pro Kit (QIAGEN, Germany). Briefly, 50–200 mg of stool was homogenised in a 2 mL bead-beating tube pre-filled with lysis beads. Mechanical disruption (bead beating) combined with chemical lysis was employed to efficiently disrupt both host and microbial cell membranes. Inhibitory substances (e.g., polysaccharides, haem compounds, and bile salts) were removed by the second-generation Inhibitor Removal Technology® (IRT). The lysate was then purified through an MB RNA Spin Column, followed by on-column DNase digestion to eliminate residual DNA. Subsequently, RNA was eluted in RNase-free water and directly used for downstream RT-qPCR analysis. The sequences of RT-qPCR primers are listed in Supplementary Table 3. The relative expression levels of each mRNA were determined by normalising to the *GAPDH* gene (for human genes), *Gapdh* (for mouse genes) and the 16S rRNA (for bacterial genes). The final results are presented as fold changes compared to the control group.

### Faecal and bacterial DNA extraction and PCR

Genomic DNA from faecal samples was extracted using the QIAamp PowerFecal Pro DNA Kit (QIAGEN, Germany) according to the manufacturer’s protocol. Briefly, 50–200 mg of stool was homogenised in lysis buffer with bead beating to ensure efficient release of bacterial DNA, followed by removal of inhibitory substances and column-based purification. For bacterial genomic DNA, engineered MG1655 strains and wild-type controls were cultured in lysogeny broth at 37 °C under aerobic conditions, harvested by centrifugation, and lysed using the TIANamp Bacteria DNA Kit (Tiangen, China). DNA quality and concentration were assessed by spectrophotometry, and purified DNA was used for downstream PCR analysis.

For quantification of target bacterial abundance in faecal samples, PCR assays were conducted using species-specific primers, with the total bacterial 16S rRNA gene serving as the internal reference. To evaluate btDPP4 expression in engineered bacterial strains, amplification results were normalised to the genomic 16S rRNA gene of MG1655. PCR reactions were performed using the same reagents and cycling conditions as described for cellular and tissue RT‑qPCR. Primer sequences are listed in Supplementary Table 3.

### Ethical statements

This study was conducted in accordance with institutional ethical guidelines and approved protocols. The use of clinical samples was approved by the Ethics Committee of the First Affiliated Hospital with Nanjing Medical University (Approval No. 2022-SR−604). All patients provided written informed consent prior to their participation. All animal experiments were carried out in accordance with the National Institutes of Health Guide for the Care and Use of Laboratory Animals. All animal experimental procedures were approved by the Institutional Animal Care and Use Committee (IACUC) of Nanjing Medical University (Approval No. IACUC−2204018).

### Statistical analysis

Statistical analysis was performed using GraphPad Prism 9.0 (GraphPad Software, USA). Data are presented as the mean ± standard deviation (SD). Student’s *t*-test or one-way ANOVA was used for normally distributed data, while the Mann–Whitney *U* test or Kruskal-Wallis test was applied for non-parametric comparisons. A *p* value <0.05 was considered statistically significant.

### Role of the funding sources

This work was supported by the National Natural Science Foundation of China (Grant No. 82370535) and the Key Discipline of Medicine in Jiangsu Province (No. ZDXK202206).

## Results

### DPP4 expression is elevated in intestinal fibro-stenotic areas of CD patients

**Figure 1.**
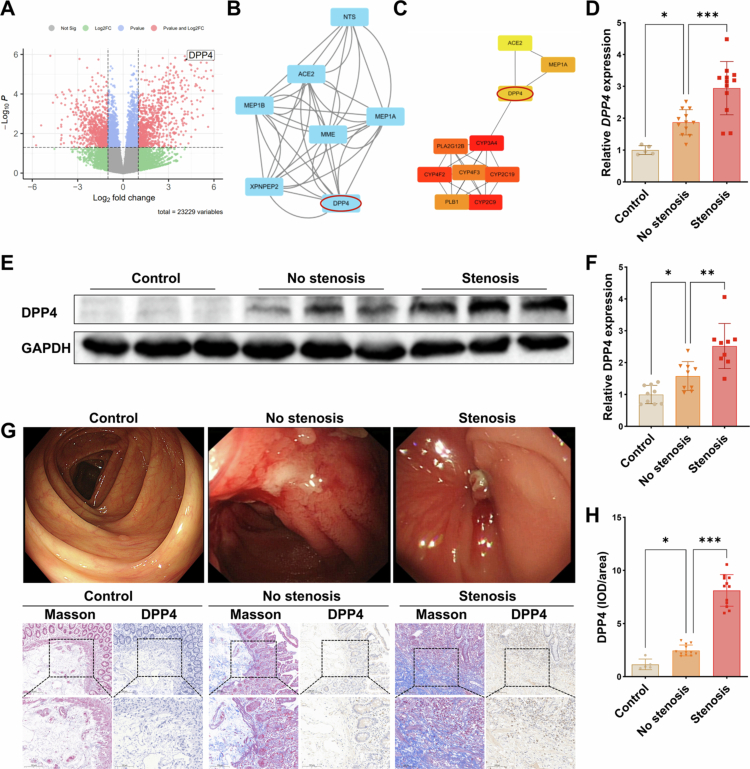
DPP4 expression is upregulated in intestinal fibro-stenotic areas of CD patients. **(A)** Volcano plot showing differentially expressed genes (DEGs) between stenotic (B2) and non-stenotic (B1) CD tissues (GSE66207 dataset). Red dots indicate DEGs with |log2FC| >1 and *p* < 0.05. **(B, C)** Hub genes identified using the MCODE and CytoHubba plugins, respectively. **(D)** RT-qPCR analysis of DPP4 mRNA levels in intestinal tissues. **(E)** Western blot analysis of DPP4 protein expression in colonic tissues from healthy controls, non-stenotic, and stenotic regions of CD patients. **(F)** Densitometric quantification of DPP4 protein normalised to GAPDH (corresponding to panel E). **(G)** Representative endoscopic images of healthy controls, non-stenotic, and stenotic intestinal regions in CD patients, illustrating macroscopic features of stricture. Corresponding immunohistochemical (IHC) images show DPP4 expression in colonic tissues from the same groups. Adjacent serial sections stained with Masson’s trichrome highlight fibrotic areas. **(H)** Quantification of IHC DPP4 staining (IOD/area) across groups. *p < 0.05; **p < 0.01; ***p < 0.001.

To identify key genes involved in intestinal stenosis in CD, we analysed a public dataset (GSE66207) containing RNA sequencing data from both stenotic (B2) and non-stenotic (B1) CD tissues. Differential expression analysis using the *limma* R package revealed 345 DEGs (|log2FC| >1, *p* < 0.05), with DPP4 among the top upregulated genes in stenotic tissues (Figure 1A). WGCNA was then applied to these DEGs to detect strongly correlated modules. The MEyellow module showed significant differences between the disease phenotypes (Figure S1A). Genes within the MEyellow module were imported into STRING for PPI analysis (Figure S1B). Subsequently, the STRING results were visualised using the Cytoscape and CytoHubba plugins to predict hub genes (Figure 1B–C). The results indicated that DPP4 was one of the top 10 hub genes, suggesting its central role in stenosis pathogenesis. To validate the differential expression of DPP4 across clinical phenotypes, we collected intestinal stenotic and non-stenotic tissues from CD patients, along with normal tissues from healthy controls. Both mRNA and protein levels of DPP4 were markedly elevated in CD tissues compared to the control group, with the highest expression observed in stenotic regions (Figure 1D–F). These findings were consistent with the sequencing data, suggesting that DPP4 may play a critical role in the fibrosis associated with CD. To assess the spatial distribution of DPP4 in relation to fibrotic remodelling, we performed immunohistochemistry (IHC) staining for DPP4 on colonic sections, in parallel with adjacent serial sections stained with Masson’s trichrome to visualise collagen deposition. While DPP4 expression was minimal in colonic tissues from healthy controls and only modestly increased in inflamed but non-stenotic CD samples, stenotic CD tissues exhibited markedly elevated DPP4 levels, with prominent localisation to stromal compartments that spatially overlapped with collagen-rich areas as confirmed by adjacent Masson’s trichrome staining (Figure 1G–H).

### Elevated DPP4 expression contributes to fibrotic remodelling in a chronic colitis model

**Figure 2.**
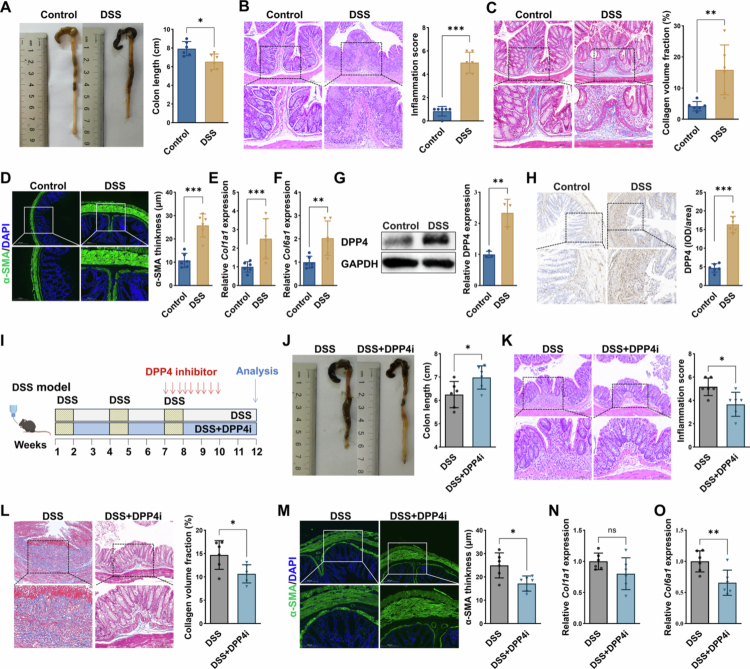
Elevated DPP4 expression contributes to fibrotic remodelling in a chronic colitis model. **(A)** Colon length quantification in control (*n* = 6) and DSS-treated (*n* = 6) mice. **(B)** Representative H&E-stained colon sections (left) and histologic inflammation scores (right). **(C)** Representative Masson’s trichrome-stained colon sections and quantification of collagen volume fraction. **(D)** Immunofluorescence images and quantification of *α*-SMA⁺ fibrotic thickness in the muscularis propria. **(E)** qRT-PCR analysis of *Col1a1* mRNA levels in colon tissues. **(F)** qRT-PCR analysis of *Col6a1* mRNA levels in colon tissues. **(G)** Western blot analysis of DPP4 protein expression in control and DSS-treated mice. **(H)** Representative IHC staining of DPP4 expression in control and DSS-treated colons, with semiquantitative analysis. **(I)** Schematic overview of the chronic DSS-induced colitis model and pharmacological intervention. Mice received three cycles of 1.5% DSS (7 days per cycle), each followed by a 14-day recovery phase. The DPP4 inhibitor sitagliptin was administered via oral gavage for 21 days during the final cycle of the experiment. **(J)** Colon length quantification in the DSS group (*n* = 6) and DSS + DPP4i group (*n* = 6). **(K)** Representative H&E-stained colon sections (left) and histologic inflammation scores (right). **(L)** Representative Masson’s trichrome-stained colon sections and quantification of collagen volume fraction. **(M)** Immunofluorescence images and quantification of *α*-SMA⁺ fibrotic thickness in the muscularis propria. **(*N*)** RT-qPCR analysis of *Col1a1* mRNA levels in colon tissues. **(O)** RT-qPCR analysis of *Col6a1* mRNA levels in colon tissues. *p < 0.05; **p < 0.01; ***p < 0.001; ns: not significant.

To clarify the role of DPP4 in intestinal fibrosis, a chronic colitis model was established using dextran sulphate sodium (DSS) to induce fibrotic remodelling in the colon. This model involved 3 cycles of 7 days of 1.5% DSS in drinking water followed by 14 days of tap water. The results showed that the colon length in the DSS group was significantly reduced (Figure 2A), and H&E staining revealed an exacerbation of inflammation, as evidenced by marked epithelial damage, crypt distortion, and inflammatory cell infiltration (Figure 2B). Masson’s trichrome staining revealed pronounced collagen deposition in the DSS group, with collagen volume fractions markedly elevated compared to the control group (Figures 2C). Immunofluorescence analysis further demonstrated submucosal fibrotic remodelling, with *α*-SMA⁺ fibrotic areas in the muscularis propria significantly thicker than controls (Figure 2D). Consistent with this, *Col1a1* and *Col6a1* mRNA levels were significantly upregulated in DSS-treated mice, confirming the establishment of intestinal fibrosis (Figure 2E–F). Importantly, both immunohistochemistry and Western blot analysis confirmed a significant increase in DPP4 expression in DSS-treated colonic tissues compared to controls, with DPP4 predominantly localised in fibrotic stromal regions (Figure 2G–H).

To further investigate the functional significance of DPP4 in intestinal fibrosis, we administered the DPP4 inhibitor (sitagliptin) via oral gavage for 21 days during the final cycle of the experiment (Figure 2I). In the DSS + DPP4i group, colon length partially recovered (Figure 2J), and the inflammation score was significantly reduced (Figure 2K), reflecting a notable attenuation of intestinal inflammation. Moreover, fibrosis in the DSS-induced chronic colitis model was alleviated, as evidenced by a reduction in collagen volume fraction (Figure 2L), and a decrease in *Col1a1* and *Col6a1* expression (Figure 2N-O). The thickness of the *α*-SMA-positive area was also significantly reduced in the DSS + DPP4i group compared to the DSS group (Figure 2M). Collectively, these findings indicate that DPP4 contributes critically to intestinal fibrogenesis, and that its pharmacological inhibition effectively suppresses fibroblast activation and fibrosis development. Importantly, sitagliptin administration was initiated only during the final DSS cycle, a time point at which chronic inflammation and tissue remodelling were already present. Therefore, the observed reduction in fibrosis-related markers under this late intervention protocol further supports a potential direct antifibrotic role of DPP4 inhibition beyond its anti-inflammatory effects.

### Membrane-bound DPP4 in fibroblasts drives intestinal myofibroblast activation and migration

**Figure 3.**
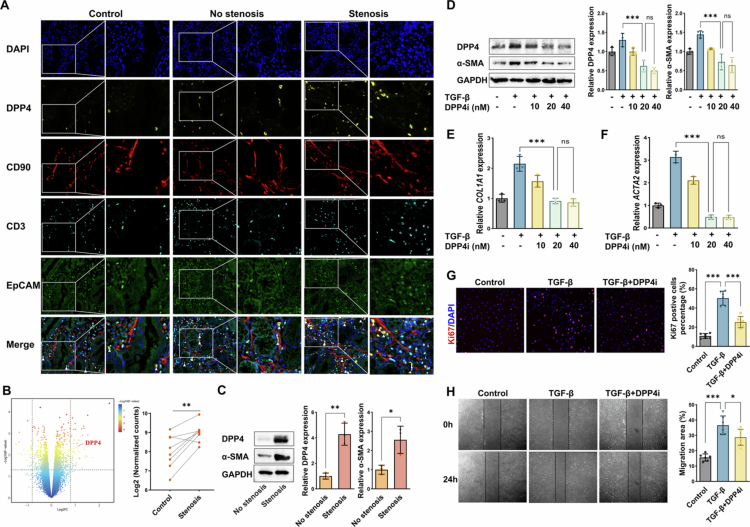
Membrane-bound DPP4 in fibroblasts drives intestinal myofibroblast activation and migration. **(A)** Multiplex IHC staining of FFPE colonic sections from healthy controls, non-stenotic, and stenotic regions of CD patients. **(B)** Volcano plot showing DEGs in fibroblasts isolated from paired stenotic and normal CD tissues (GSE90607 dataset, left), with *DPP4* mRNA expression levels specifically highlighted (right). **(C)** Western blot analysis of membrane-bound DPP4 and *α*-SMA protein in primary HIMFs. **(D)** Western blot analysis and quantification of DPP4 and *α*-SMA expression in TGF-β–stimulated HIMFs with or without DPP4 inhibitor. **(E)** qRT-PCR analysis of *COL1A1* mRNA expression in HIMFs. **(F)** qRT-PCR analysis of *ACTA2* mRNA expression in HIMFs. **(G)** Immunofluorescence staining and quantification of Ki67⁺ proliferating HIMFs. **(H)** Representative images and quantification of HIMF migration in scratch wound healing assays. *p < 0.05; **p < 0.01; ***p < 0.001; ns: not significant.

To examine DPP4 localisation in intestinal tissues, we conducted multiplex immunohistochemistry (mIHC) on FFPE sections from controls, non-stenotic, and stenotic CD tissues. Co-staining with CD3 (T cells), EpCAM (epithelial cells), and CD90 (fibroblasts) revealed that DPP4 predominantly co-localised with CD90⁺ cells, but not with CD3⁺ or EpCAM⁺ cells. This suggests that DPP4 is primarily expressed by intestinal fibroblasts. Moreover, DPP4⁺CD90⁺ fibroblasts were markedly enriched in fibrotic regions of stenotic CD tissues compared to non-fibrotic and control samples (Figure 3A). Transcriptomic analysis of the GSE90607 dataset—comprising paired fibroblasts isolated from stenotic and normal CD tissues—further confirmed elevated *DPP4* mRNA levels in stenotic fibroblasts (Figure 3B). To further support the presence of membranous DPP4 on fibroblasts, we performed flow cytometry analysis on live, non-permeabilized HIMFs using surface DPP4 antibodies. The results revealed a distinct DPP4⁺ population, confirming the existence of membrane-bound DPP4 on intestinal fibroblasts (Figure S2). To validate these findings, we isolated primary myofibroblasts from stenotic and non-stenotic intestinal regions and analysed membrane-bound DPP4 protein levels. Western blot confirmed significantly increased DPP4 levels in HIMFs from stenotic compared to non-stenotic regions (Figure 3C).

To explore the functional role of membrane-bound DPP4 in intestinal fibrosis, we performed *in vitro* assays using HIMFs. Myofibroblast activation was first induced by TGF-*β* treatment and key biological processes such as proliferation, migration, and extracellular matrix (ECM) deposition were assessed. TGF-*β* stimulation significantly upregulated membrane-bound DPP4 protein levels. Notably, pharmacological inhibition of DPP4 with sitagliptin (DPP4i) attenuated myofibroblast activation in a dose-dependent manner, with maximal *α*-SMA suppression observed at 20 nM (Figure 3D). This concentration was therefore selected for subsequent experiments. Consistent with phenotypic reversal, DPP4i downregulated mRNA levels of the fibrotic markers *COL1A1* and *ACTA2* (Figure 3E–F). Proliferation assays revealed that DPP4i significantly reduced the proportion of Ki67⁺ cells (Figure 3G). Similarly, scratch wound healing assays demonstrated that DPP4i abrogated TGF-*β*-induced migration, significantly decreasing wound closure rates (Figure 3H). These data collectively demonstrate that membrane-bound DPP4 drives fibroblast activation, proliferation, and migration, establishing its pivotal role in intestinal fibrogenesis.

### Soluble DPP4 (sDPP4) promotes intestinal myofibroblast activation, proliferation, and migration

**Figure 4.**
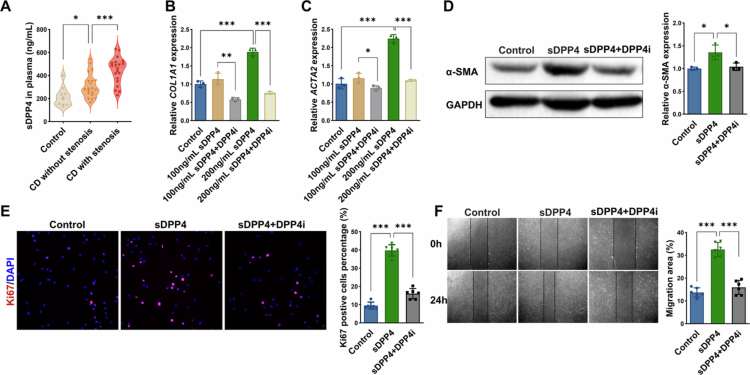
Soluble DPP4 (sDPP4) promotes intestinal myofibroblast activation, proliferation, and migration. **(A)** ELISA-based quantification of soluble DPP4 (sDPP4) levels in plasma from healthy controls (*n* = 10), CD patients without stenosis (*n* = 20), and with stenosis (*n* = 20). **(B)** RT-qPCR analysis of *COL1A1* mRNA expression in HIMFs treated with sDPP4 in the presence or absence of DPP4 inhibitor. **(C)** RT-qPCR analysis of *ACTA2* mRNA expression in HIMFs treated with sDPP4 in the presence or absence of DPP4 inhibitor. **(D)** Western blot of *α*-SMA protein expression in HIMFs treated with sDPP4 in the presence or absence of DPP4 inhibitor. **(E)** Immunofluorescence staining and quantification of Ki67⁺ proliferating HIMFs treated with sDPP4 in the presence or absence of DPP4 inhibitor. **(F)** Scratch wound healing assay evaluating HIMF migration at 0 and 24 hours following sDPP4 stimulation, with or without DPP4 inhibitor. Wound closure was quantified using ImageJ. *p < 0.05; **p < 0.01; ***p < 0.001.

Beyond membrane-bound DPP4, we also investigated the profibrotic effects of soluble DPP4 (sDPP4) on human intestinal myofibroblasts (HIMFs). First, we measured sDPP4 concentrations in plasma from healthy controls, CD patients without stenosis, and CD patients with stenosis (CDAI-matched to the non-stenotic group). Plasma sDPP4 levels were modestly elevated in non-stenotic CD patients compared to healthy controls and significantly higher in stenotic CD patients (Figure 4A), suggesting a potential role for sDPP4 in fibrosis progression independent of inflammation. To assess the functional impact of sDPP4, HIMFs were treated with recombinant sDPP4 (100 ng/mL or 200 ng/mL), with or without the DPP4 inhibitor sitagliptin (20 nM). sDPP4 stimulation increased the expression of fibrotic markers in a concentration-dependent manner, with stronger effects observed at 200 ng/mL. At this concentration, both *COL1A1* and *ACTA2* mRNA levels were significantly elevated relative to controls, and this induction was attenuated by DPP4 inhibition (Figure 4B–C). Western blot analysis confirmed that sDPP4 (200 ng/mL) elevated *α*-SMA protein levels, indicative of myofibroblast activation (Figure 4D). Proliferation assays revealed that sDPP4 increased the proportion of Ki67⁺ proliferating cells, and this effect was reversed by sitagliptin (Figure 4E). Similarly, scratch wound healing assays demonstrated that sDPP4 enhanced HIMF migration and increased wound closure rates, which was suppressed by DPP4 inhibition (Figure 4F). These findings suggest that sDPP4 may synergise with membrane-bound DPP4 to amplify fibrogenic responses, underscoring the therapeutic relevance of targeting both forms of DPP4 in CD-associated fibrosis.

### DPP4 drives intestinal myofibroblast activation via the PI3K-AKT pathway

**Figure 5.**
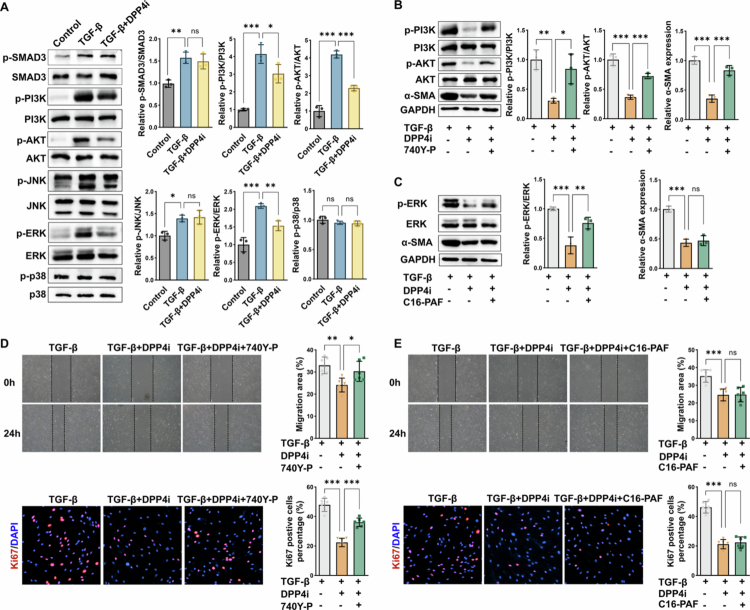
DPP4 drives intestinal myofibroblast activation via the PI3K-AKT pathway. **(A)** Western blot analysis of canonical (SMAD3) and non-canonical (PI3K-AKT, ERK, JNK, and p38) signalling pathways in TGF-*β*-stimulated HIMFs, treated with or without DPP4 inhibitor (sitagliptin, 20 nM). **(B)** Western blot analysis of *p*-PI3K, PI3K, *p*-AKT, AKT, and *α*-SMA expression in HIMFs treated with TGF-*β*, in the presence or absence of DPP4 inhibitor (20 nM) and the PI3K activator 740Y-*P* (10 μM). **(C)** Western blot analysis of *p*-ERK, ERK, and *α*-SMA expression in HIMFs treated with TGF-*β*, in the presence or absence of DPP4 inhibitor (20 nM) and the MEK/ERK activator C16-PAF (1 μM). **(D)** Scratch wound healing assay of HIMF migration at 0 and 24 hours following TGF-*β* stimulation with or without DPP4 inhibitor (20 nM) and the PI3K activator 740Y-*P* (10 μM). Wound closure was quantified using ImageJ. Ki67 immunofluorescence staining and quantification of proliferating HIMFs are shown in the lower panels. **(E)** Scratch wound healing assay of HIMF migration at 0 and 24 hours following TGF-*β* stimulation with or without DPP4 inhibitor (20 nM) and the MEK/ERK activator C16-PAF (1 μM). Wound closure was quantified using ImageJ. Ki67 immunofluorescence staining and quantification are shown in the lower panels. *p < 0.05; **p < 0.01; ***p < 0.001; ns: not significant.

To elucidate how DPP4 activates myofibroblasts, we first analysed a single-cell RNA-seq dataset of stenotic intestinal tissues from CD patients.^13^ Fibroblasts were classified into DPP4⁺ and DPP4⁻ subsets, followed by differential gene expression and KEGG pathway enrichment analysis. This analysis revealed significant enrichment of both the canonical TGF-*β* signalling pathway and the non-canonical PI3K–AKT pathway among the DEGs (Figure S3), indicating that DPP4⁺ fibroblasts transcriptionally engage profibrotic signalling programs. Guided by these transcriptomic findings, we next investigated the downstream signalling mechanisms through which DPP4 promotes fibroblast activation in primary HIMFs. TGF-*β* activates fibroblasts through both canonical (SMAD3-dependent) and non-canonical pathways, including p38 mitogen-activated protein kinase (MAPK), extracellular signal-regulated kinase (ERK), JUN *N*-terminal kinase (JNK), and the phosphoinositide 3-kinase (PI3K)-AKT cascades.^19^ Western blot analysis showed that DPP4 inhibition with sitagliptin (20 nM) significantly reduced the phosphorylation of PI3K, AKT, and ERK, while the total protein levels of these kinases remained unchanged (Figure 5A). To test pathway specificity, we performed rescue experiments using a PI3K activator (740Y-*P*, 10 μM) and a MEK/ERK activator (C16-PAF, 1 μM). Co-treatment with 740Y-*P* reversed DPP4i-induced suppression of *α*-SMA expression, whereas C16-PAF showed no significant effect (Figure 5B–C). Functional assays further supported the specificity of the PI3K-AKT pathway: 740Y-*P* significantly restored HIMF migration and proliferation suppressed by DPP4 inhibition, whereas C16-PAF showed no such effect (Figure 5D–E). Collectively, these findings identify the PI3K-AKT axis, rather than ERK signalling, as the principal downstream effector mediating DPP4-induced intestinal myofibroblast activation.

### Gut microbiota-derived DPP4 is enriched in stenotic CD and associated with fibrotic remodelling

**Figure 6.**
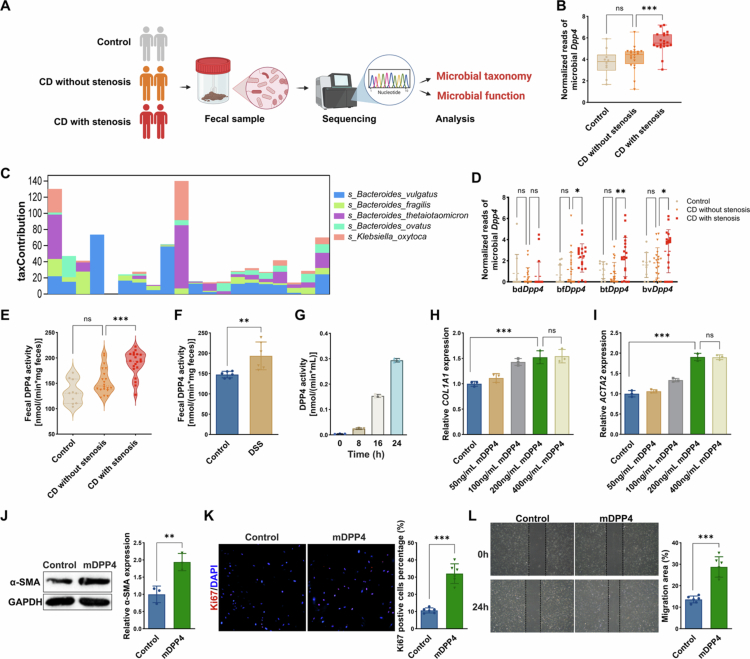
Gut microbiota-derived DPP4 is enriched in stenotic CD and associated with fibrotic remodelling. **(A)** Schematic overview of the faecal metagenomic sequencing strategy in controls (*n* = 10), CD without stenosis (*n* = 20), and CD with stenosis (*n* = 20). **(B)** Relative abundance of microbial *Dpp4* genes across the three groups, as determined by metagenomic sequencing. **(C)** Species-level contributions of microbial *Dpp4* genes in CD patients with stenosis, showing the top five contributing species ranked by relative abundance. **(D)** Differential abundance of microbial Dpp4 genes assigned to four Bacteroides species in faecal metagenomes from healthy controls, non-stenotic CD, and stenotic CD patients. **(E)** Total faecal DPP4 enzymatic activity was measured by Gly-Pro-pNA assay in controls, CD patients without stenosis, and those with stenosis. **(F)** Faecal DPP4 enzymatic activity was measured in control mice and those with DSS-induced chronic colitis. **(G)** Time-dependent increase in DPP4 activity measured in the culture supernatants of *B. thetaiotaomicron* grown under anaerobic conditions. **(H–I)** RT-qPCR analysis of *COL1A1* (H) and *ACTA2* (I) mRNA levels in HIMFs treated with increasing concentrations of recombinant btDPP4 (0–400 ng/mL). **(J)** Western blot analysis of *α*-SMA protein expression in HIMFs treated with 200 ng/mL btDPP4. **(K)** Ki67 immunofluorescence staining and quantification of proliferating HIMFs following treatment with 200 ng/mL btDPP4. **(L)** Scratch wound healing assay of HIMF migration at 0 and 24 hours following treatment with 200 ng/mL btDPP4. *p < 0.05; **p < 0.01; ***p < 0.001; ns: not significant.

In addition to host-derived DPP4, microbial factors have garnered increasing recognition as key contributors to the pathophysiology of CD. Prompted by reports that specific gut microbes secrete enzymatically active DPP4, we hypothesised that microbiota-derived DPP4 may also promote intestinal fibrosis in CD.^20^ To investigate this possibility, we conducted metagenomic sequencing on faecal samples from 50 individuals, including healthy controls (*n* = 10), non-stenotic CD patients (*n* = 20), and stenotic CD patients (*n* = 20) (Figure 6A). Taxonomic and functional analysis indicated a non-significant trend toward increased microbial *Dpp4* abundance in non-stenotic CD patients. By contrast, microbial *Dpp4* levels were significantly elevated in stenotic CD patients compared to both non-stenotic CD and healthy controls (Figure 6B). Microbial DPP4 homologues were predominantly encoded by members of the genus *Bacteroides*, particularly *B. dorei*, *B. fragilis*, *B. thetaiotaomicron*, and *B. vulgatus* (Figure 6C, Figure S4A). Among these, bt*Dpp4* showed the most pronounced differential abundance between CD patients with stenosis and those without (Figure 6D, Figure S4B). These findings were further supported by RT-qPCR analysis, which revealed a comparable pattern of microbial *Dpp4* mRNA expression (Figure S4C). In parallel, chronic DSS-treated mice also demonstrated significantly increased faecal microbial *Dpp4* expression compared to controls (Figure S4D). To further evaluate enzymatic activity, total faecal DPP4 activity was measured across all three groups. Faecal DPP4 activity was significantly elevated in stenotic CD patients relative to both non-stenotic CD and healthy controls (Figure 6E). This pattern was mirrored in DSS-induced fibrotic mice (Figure 6F).

Given its substantial species-level contribution to microbiota-derived DPP4 and the marked enrichment of btDPP4 in stenotic CD patients, *B. thetaiotaomicron* was selected as a representative microbe for downstream validation. Whole-genome analysis confirmed the presence of a conserved *Dpp4*-encoding gene in its genome (Figure S4E). Moreover, when cultured under anaerobic conditions, *B. thetaiotaomicron* secretes DPP4 into the extracellular environment in a time-dependent manner, as evidenced by increasing DPP4 activity in the culture supernatant (Figure 6G, Figure S4F–G). These findings demonstrate that microbial DPP4 is not only present but also actively secreted and enzymatically functional.

To functionally validate its fibrogenic effects, recombinant *B. thetaiotaomicron*-derived DPP4 (btDPP4) was synthesised and applied to primary HIMFs. BtDPP4 dose-dependently upregulated *COL1A1* and *ACTA2* mRNA levels, with maximal induction observed at 200 ng/mL (Figure 6H–I). At this dose, btDPP4 significantly increased *α*-SMA protein expression and the proportion of Ki67⁺ proliferating cells (Figure 6J–K). Moreover, btDPP4 treatment enhanced HIMF migration as assessed by scratch wound healing assays (Figure 6L). Collectively, these data identify microbiota-derived DPP4, particularly from *B. thetaiotaomicron*, as a direct and functional contributor to fibroblast activation and intestinal fibrogenesis in CD.

### Colonisation with engineered bacteria overexpressing DPP4 worsens fibrotic remodelling in DSS-induced chronic colitis

**Figure 7.**
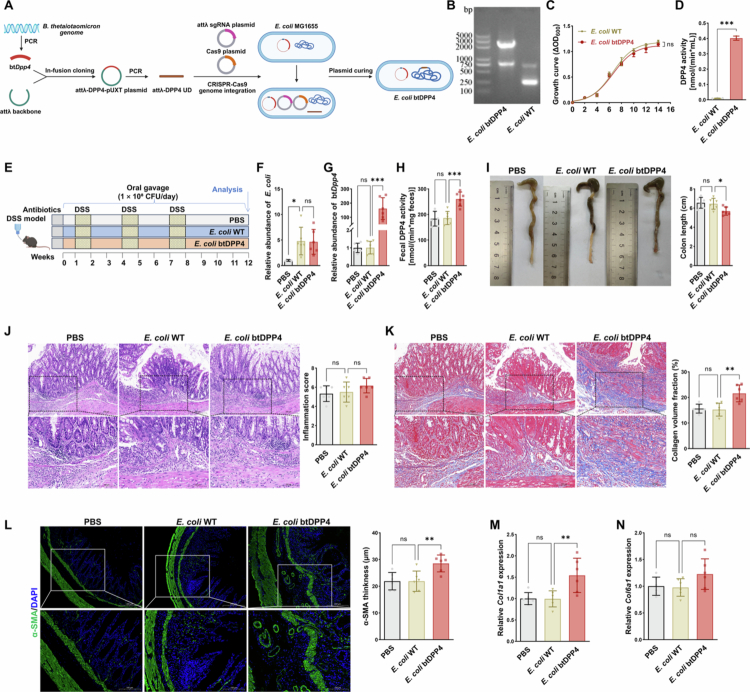
Colonisation with engineered bacteria overexpressing DPP4 worsens fibrotic remodelling in DSS-induced chronic colitis. **(A)** Schematic illustration of engineered *E. coli* btDPP4 construction. **(B)** PCR validation of btDPP4 genomic integration in engineered *E. coli* strains. **(C)** Growth curves comparing *E. coli* btDPP4 and wild-type (WT) strains under standard conditions. **(D)** Secreted DPP4 enzymatic activity measured in the culture supernatants of *E. coli* btDPP4 and *E. coli* WT. **(E)** Experimental design of the chronic DSS colitis model with oral gavage of PBS, *E. coli* WT, or *E. coli* btDPP4. **(F)** Relative faecal *E. coli* load in mice treated with PBS, *E. coli* WT, or *E. coli* btDPP4. **(G)** Expression of btDPP4 gene in faeces from mice treated with PBS, *E. coli* WT, or *E. coli* btDPP4. **(H)** Faecal DPP4 enzymatic activity in mice treated with PBS, *E. coli* WT, or *E. coli* btDPP4. **(I)** Colon length was measured and compared across groups. **(J)** Representative H&E-stained colon sections and quantification of histological inflammation scores. **(K)** Representative Masson’s trichrome-stained sections and quantification of collagen volume fraction. **(L)** Immunofluorescence staining of *α*-SMA⁺ fibrotic areas and quantification of fibrotic thickness. **(M–N)** RT-qPCR analysis of *Col1a1* (M) and *Col6a1* (*N*) mRNA levels in mouse colon tissues. *p < 0.05; **p < 0.01; ***p < 0.001; ns: not significant.

To further clarify the *in vivo* contribution of microbiota-derived DPP4 to fibrotic remodelling, we generated an engineered *E. coli* strain overexpressing btDPP4 (*E. coli* btDPP4) (Figure 7A). Overexpression of btDPP4 in *E. coli* btDPP4 was validated by PCR and Western blotting (Figure 7B; Figure S5A–B). This strain displayed comparable growth kinetics to *E. coli* WT; however, it exhibited markedly higher extracellular DPP4 enzymatic activity, confirming functional overexpression (Figure 7C–D). In the chronic DSS colitis model, oral administration of either *E. coli* WT or *E. coli* btDPP4 led to markedly increased faecal *E. coli* abundance compared with PBS-treated controls. Notably, mice receiving *E. coli* btDPP4 showed higher btDPP4 gene copy numbers and elevated faecal DPP4 enzymatic activity compared to those colonised with *E. coli* WT, confirming stable colonisation and functional expression of the engineered strain (Figure 7F–H). Consistent with this colonisation, mice receiving *E. coli* btDPP4 displayed exacerbated fibrotic remodelling, including pronounced colon shortening (Figure 7I) and increased collagen deposition on Masson’s trichrome staining (Figure 7J–K). Histological inflammation scores showed a mild, non-significant increase in mice treated with *E. coli* btDPP4. Moreover, mice colonised with *E. coli* btDPP4 displayed increased stromal *α*-SMA thickening, along with upregulated *Col1a1* and *Col6a1* expression compared to the *E. coli* WT group (Figure 7L–N). Collectively, these results demonstrate that microbiota-derived DPP4 exacerbates intestinal fibrosis in the DSS-induced chronic colitis model.

### Microbial-derived DPP4 inhibitor Dau-d4 suppresses intestinal myofibroblast activation

**Figure 8.**
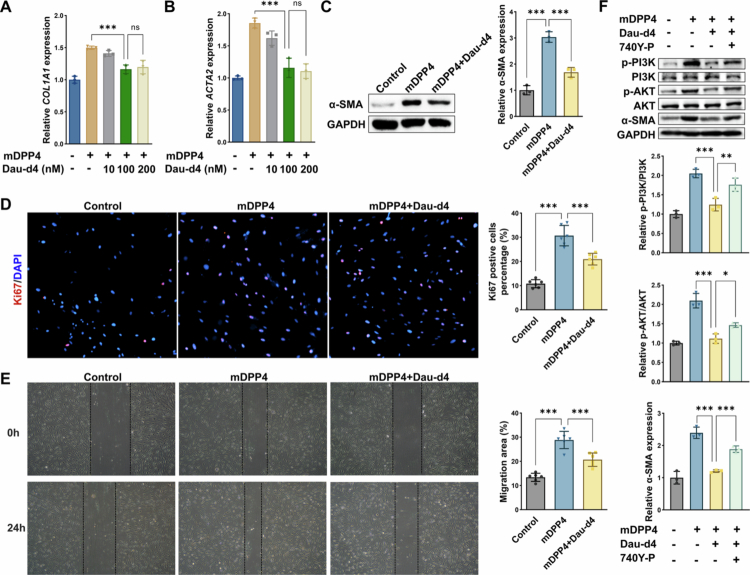
Microbial-derived DPP4 inhibitor Dau-d4 suppresses intestinal myofibroblast activation. **(A-B)** RT-qPCR analysis of *COL1A1* and *ACTA2* mRNA expression in HIMFs treated with increasing concentrations of recombinant microbial DPP4 (btDPP4, hereafter referred to as mDPP4), with or without the microbial DPP4 inhibitor Dau-d4 (0–200 nM). **(C)** Western blot analysis of *α*-SMA protein expression in HIMFs stimulated with mDPP4, with or without 100 nM Dau-d4. **(D)** Ki67 immunofluorescence staining and quantification of proliferating HIMFs stimulated with mDPP4, with or without 100 nM Dau-d4. **(E)** Scratch wound healing assay showing HIMF migration at 0 and 24 hours following treatment with mDPP4, in the presence or absence of 100 nM Dau-d4. **(F)** Western blot analysis of *p*-PI3K, PI3K, *p*-AKT, AKT, and *α*-SMA expression in HIMFs treated with vehicle control, mDPP4 (200 ng/mL), mDPP4 + Dau-d4 (100 nM), or mDPP4 + Dau-d4 + PI3K activator 740Y-*P* (10 μM) for 24 hours. *p < 0.05; **p < 0.01; ***p < 0.001; ns: not significant.

To assess the therapeutic potential of targeting microbiota-derived DPP4, we utilised Dau-d4, a microbial-selective DPP4 inhibitor with no cross-reactivity to the human enzyme. Dau-d4 treatment dose-dependently attenuated btDPP4-induced upregulation of fibrotic markers in HIMFs, with maximal inhibition of *COL1A1* and *ACTA2* mRNA levels observed at 100 nM (Figure 8A–B). At 100 nM, Dau-d4 significantly suppressed btDPP4-induced increase in *α*-SMA protein expression (Figure 8C) and reduced the proportion of Ki67⁺ proliferating HIMFs (Figure 8D). Scratch wound healing assays further showed that Dau-d4 abrogated btDPP4-induced HIMF migration and significantly reduced wound closure rates (Figure 8E). These findings highlight the efficacy of Dau-d4 in blocking microbiota-driven fibrogenic responses, supporting its potential as a therapeutic strategy for CD-associated intestinal fibrosis. To further elucidate the downstream signalling mechanisms of microbiota-derived DPP4, we assessed PI3K-AKT pathway activation in HIMFs following mDPP4 stimulation. Western blot analysis revealed that mDPP4 treatment markedly enhanced phosphorylation of PI3K and AKT, and concurrently upregulated *α*-SMA protein expression. These effects were significantly attenuated by co-treatment with Dau-d4. Notably, the addition of 740Y-*P* to the Dau-d4-treated cells partially restored phosphorylation of PI3K/AKT and *α*-SMA expression (Figure 8F). These findings indicate that microbiota-derived DPP4 promotes myofibroblast activation predominantly through the PI3K-AKT pathway, mirroring the mechanism observed for host DPP4 signalling.

### Dual inhibition of bacterial- and host-derived DPP4 synergistically attenuates intestinal fibrosis *in vivo*

**Figure 9.**
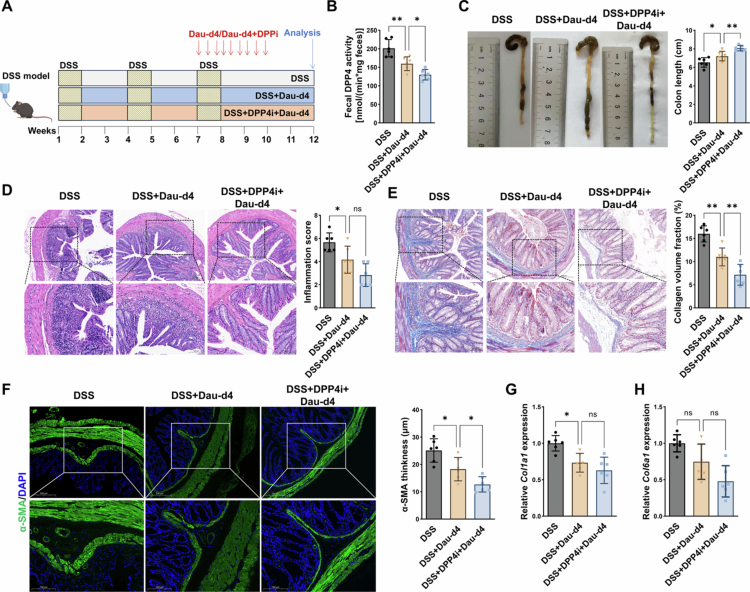
Dual inhibition of bacterial- and host-derived DPP4 synergistically attenuates intestinal fibrosis *in vivo.* **(A)** Schematic of the experimental design showing late-phase intervention with the microbiota-derived DPP4 inhibitor Dau-d4 (10 mg/kg), alone or in combination with the host-derived DPP4 inhibitor sitagliptin, during the final cycle of DSS-induced chronic colitis. **(B)** DPP4 activity was measured in the murine faeces following treatment. **(C)** Colon length was measured and compared across groups. **(D)** Representative H&E-stained colon sections and quantification of histological inflammation scores. **(E)** Representative images of Masson’s trichrome staining and quantification of collagen volume fraction. **(F)** Immunofluorescence staining of *α*-SMA⁺ areas and quantification. **(G-H)** RT-qPCR analysis of *Col1a1* and *Col6a1* mRNA expression in murine colonic tissues. *p < 0.05; **p < 0.01; ***p < 0.001; ns: not significant.

To further assess the contribution of bacterial and host-derived DPP4 to intestinal fibrosis in CD, and to evaluate the therapeutic potential of targeting both sources of DPP4, we conducted *in vivo* experiments using the DSS-induced chronic colitis model. During the final cycle of the model, we administered the bacterial-derived DPP4 inhibitor Dau-d4 (10 mg/kg), either alone or in combination with the human DPP4 inhibitor sitagliptin (Figure 9A). Faecal DPP4 activity was subsequently measured to assess enzymatic inhibition. The results demonstrated that Dau-d4 significantly suppressed faecal DPP4 enzymatic activity, confirming its *in vivo* efficacy (Figure 9B). Notably, treatment with Dau-d4 led to a partial recovery of intestinal length (Figure 9C) and a reduction in inflammatory infiltration as shown by H&E staining (Figure 9D). Masson’s trichrome staining showed a significant decrease in collagen volume fraction within the intestinal submucosa (Figure 9E). Immunofluorescence staining further confirmed reduced *α*-SMA-positive areas, indicative of attenuated submucosal fibrosis (Figure 9F). Importantly, co-administration of Dau-d4 and sitagliptin resulted in further improvements in multiple fibrosis-related parameters, including intestinal length, inflammatory infiltration, collagen deposition, and *α*-SMA thickness (Figure 9C–F). Moreover, this combination more effectively reduced the expression of fibrotic genes, including *Col1a1* and *Col6a1*, compared to Dau-d4 treatment alone (Figure 9G–H). These findings demonstrate that targeting bacterial-derived DPP4 effectively mitigates intestinal fibrosis in CD, and that dual inhibition of both microbial and host DPP4 may yield even more pronounced therapeutic benefits.

## Discussion

CD is a chronic, progressive, and immune-mediated disorder characterised by alternating periods of remission and relapse. Intestinal fibrosis is a frequent and debilitating complication of CD, often necessitating surgical intervention and significantly impairing patient quality of life. Despite extensive research efforts, the mechanisms underlying the formation of intestinal fibrosis in CD remain poorly understood. The interplay between inflammation and fibrosis has long been a subject of intense investigation. Fibrosis was traditionally viewed as an irreversible process triggered by inflammation.^3^ However, accumulating clinical evidence challenges this paradigm, as anti-inflammatory therapies often fail to halt fibrotic progression in CD patients.^21^^,^^22^ This discrepancy has led to growing recognition that intestinal fibrosis is not simply a downstream consequence of inflammation, but rather an active, self-sustaining, and potentially reversible process governed by distinct molecular mechanisms.^23^

To uncover potential therapeutic targets for fibrosis reversal, we analysed GEO datasets comparing stenotic and non-stenotic intestinal tissues from CD patients, which identified DPP4 as a prominent hub gene associated with fibrotic remodelling. We further validated that both membrane-bound and soluble forms of DPP4 (sDPP4) were significantly upregulated in fibrotic intestinal regions from CD patients with strictures. Similarly, in a DSS-induced chronic colitis model, fibrosis formation was accompanied by increased DPP4 protein expression. Importantly, pharmacological inhibition of DPP4 using sitagliptin significantly reduced collagen deposition, *α*-SMA expression, and fibroblast migration both *in vitro* and *in vivo*, supporting a functional, rather than merely correlative, role for DPP4 in intestinal fibrosis. Beyond its well-established role in type 2 diabetes, where it regulates GLP−1 metabolism and insulin secretion, DPP4 has also been implicated in the development of fibrosis across multiple organs, including the liver, kidney, and skin.^9^^,^^11^ However, its role in intestinal fibrosis remains largely unexplored. Our findings highlight the importance of DPP4 as a therapeutic target in intestinal fibrosis and provide mechanistic insights into its profibrotic role in CD. While DPP4 inhibitors are clinically deployed for diabetes, our data expand their therapeutic relevance to CD-related intestinal stricture, a concept supported by preclinical studies in scleroderma, diabetic nephropathy, pulmonary and hepatic fibrosis.^11^^,^^24^^,^^25^

Given the dual existence of DPP4 in membrane-bound and soluble forms, we examined their respective contributions to intestinal fibrosis. Activated myofibroblasts, key drivers of fibrogenesis, exhibited enriched co-expression of DPP4 and CD90 in stenotic CD tissues. Using primary HIMFs, we demonstrated that membrane-bound DPP4 promotes fibroblast-to-myofibroblast transition and ECM deposition. Pharmacological inhibition of DPP4 attenuated HIMF migration and *α*-SMA expression, underscoring its role in cytoskeletal remodelling. Together, these spatial localisation and functional data strongly suggest a cell-autonomous role of DPP4 in driving fibrogenesis. This aligns with studies in systemic sclerosis that identify DPP4 as a marker of activated dermal fibroblasts, suggesting conserved fibrogenic mechanisms across tissues.^26^ Future genetic studies, utilising conditional knockout of DPP4 specifically in fibroblasts or ablation of DPP4-positive fibroblasts, will be valuable to formally confirm this mechanism *in vivo* and exclude potential contributions from other cell types.

In parallel, plasma levels of soluble DPP4 (sDPP4) were elevated in stenotic CD patients, independent of disease activity. Functional assays further demonstrated that sDPP4 promotes HIMF proliferation, activation, and migration. Casrouge et al. reported that lymphocytes are a major source of circulating sDPP4,^27^with Th17 cells exhibiting the highest DPP4 expression among CD4⁺ T cell subsets.^28^ Given that Th17 cells can release sDPP4 and play a pivotal role in CD-associated intestinal fibrosis, they are likely a major source of sDPP4 in stenotic CD intestines.^18^^,^^27^^,^^29^ Mechanistically, our data reveal that DPP4-mediated fibroblast activation primarily proceeds via the PI3K-AKT pathway. Although DPP4 inhibition downregulated both *p*-AKT and *p*-ERK in TGF-β–stimulated HIMFs, only PI3K-AKT reactivation restored *α*-SMA expression and fibrotic phenotypes. In contrast, ERK activation had no significant effect, suggesting a subordinate or compensatory role for ERK signalling in DPP4-mediated fibroblast activation. These observations are consistent with previous reports indicating that reduced DPP4 activity attenuates PI3K signalling in inflamed colonic tissues.^30^ Although ERK activation alone was insufficient, potential crosstalk between PI3K and ERK cannot be ruled out and warrants further investigation in future studies employing combined pathway modulation. Notably, DPP4 inhibitors have been reported to induce conformational changes in DPP4, suggesting that their effects extend beyond merely neutralising sDPP4.^31‐33^ Current studies have shown an increased risk of diabetes in patients with IBD,^34^but clinical data on concurrent use of DPP4 inhibitors in CD remain limited. Importantly,DPP4 inhibitors do not reduce blood glucose under normoglycemic conditions,thereby minimising the risk of hypoglycemia and supporting their potential as a safe therapeutic strategy for CD-associated fibrosis.^35^

Beyond host-expressed DPP4, our study uncovers a previously unrecognised role for gut microbiota-derived DPP4 in CD-associated intestinal fibrogenesis. Metagenomic sequencing revealed a significant enrichment of microbiota-derived DPP4 in the faeces of stenotic CD patients. This upregulation remained evident when comparing CDAI-matched CD patients with and without fibrosis, further supporting a fibrosis-associated role for microbiota-derived DPP4. Notably, *Bacteroides* emerged as the predominant genus encoding microbial *Dpp4* in our dataset, suggesting that specific commensal taxa may act as microbial reservoirs for bioactive molecules with fibrogenic potential. Although studies have reported variable changes in *Bacteroides* abundance among CD cohorts,^36‐38^ such compositional differences do not necessarily reflect functional capacity. The *Dpp4* gene exhibits functional redundancy across multiple commensal genera, which may collectively maintain the microbial DPP4 potential in fibrostenotic CD. Also, *Bacteroides* strains with high DPP4 expression may be selectively enriched at fibrotic sites, which are not fully captured by faecal analysis. Moreover, DPP4 is a secreted enzyme whose expression, release, and activity can be modulated by local inflammatory and metabolic cues, indicating that its fibrogenic effect depends on overall function rather than the abundance of any single genus. Together, these mechanisms support the concept that microbial DPP4 functions as a community-level and compartment-specific mediator, linking dysbiosis to fibrostenotic remodelling in CD. Consistent with this notion, functional assays further demonstrated the profibrotic effects of microbial DPP4, providing direct experimental support for its pathogenic potential. Recombinant microbial DPP4 dose-dependently induced fibroblast activation *in vitro*, while oral administration of engineered *E. coli* overexpressing microbial DPP4 exacerbated intestinal fibrosis *in vivo*. Although the recombinant DPP4 used in this study was derived from *Bacteroides thetaiotaomicron* (due to its high taxonomic contribution), and may not fully represent the complexity of microbiota-derived DPP4, our findings demonstrate that even this single-species source can drive fibrotic remodelling. These results establish a host-microbiota synergy wherein both compartments converge on a shared fibrogenic pathway. Mechanistically, we further demonstrated that microbial-derived DPP4 also activates the PI3K-AKT signalling pathway in HIMFs, as evidenced by increased *p*-PI3K, *p*-AKT and *α*-SMA levels upon btDPP4 stimulation. These effects were attenuated by the microbial DPP4 inhibitor Dau-d4, and partially rescued by the PI3K activator 740Y-*P*, confirming that mDPP4 promotes fibrotic activation predominantly through this pathway. While the PI3K-AKT axis appears to be the primary downstream mediator, additional microbe–host interaction pathways may also contribute and warrant further investigation.

The contribution of gut microbiota to CD-associated fibrosis has gained increasing attention, with recent multi-omics studies identifying microbial metabolites as key modulators of fibrogenesis. For example, Xu et al. reported that adherent-invasive *Escherichia coli* (AIEC), a CD-associated pathobiont, downregulates let-7b expression in intestinal epithelial cell-derived exosomes, thereby modulating macrophage polarisation and exacerbating intestinal fibrosis.^6^ Similarly, Li et al. performed an integrative multi-omics analysis combining gut microbiota profiling, faecal and plasma metabolomics, and magnetic resonance enterography (MRE), and identified specific bacterial taxa (e.g., *Lachnospiraceae*, *Ruminococcaceae*) and metabolites (e.g., L-aspartic acid, glutamine) associated with moderate-to-severe intestinal fibrosis​.^39^ Their findings suggest that microbial metabolic activity contributes to fibrosis progression. These findings align with our hypothesis that microbial components, including enzymatic factors such as DPP4, directly contribute to fibroblast activation and ECM deposition. Current limitations in microbial culturing and metagenomic annotation impede comprehensive characterisation of DPP4-producing bacterial taxa. Future studies utilising gnotobiotic models or species-specific DPP4 knockouts may dissect the contributions of additional taxa, such as *Clostridium* or *Escherichia*. Nevertheless, our work underscores the therapeutic potential of targeting microbial DPP4, particularly in conjunction with host-directed therapies, to disrupt this cooperative fibrotic network.

To extend our mechanistic insights to clinical relevance, we evaluated the efficacy of Dau-d4, a selective inhibitor of microbiota-derived DPP4, in a DSS-induced chronic colitis model. Dau-d4 monotherapy significantly attenuated intestinal fibrosis, reducing collagen deposition and *α*-SMA⁺ areas compared to DSS controls. Strikingly, co-treatment with Dau-d4 and the human DPP4 inhibitor sitagliptin further normalised colon length and more potently suppressed *Col1a1* and *Col6a1* expression, demonstrating synergistic antifibrotic efficacy. Notably, in our chronic colitis model, both sitagliptin and Dau-d4 were administered only during the final DSS cycle, at a stage when fibrotic remodelling was already established and inflammation had transitioned into a chronic phase. Even if DPP4 inhibition exerts some anti-inflammatory effects, the timing of intervention renders such effects insufficient to account for the observed antifibrotic outcomes. This late-phase intervention therefore implies that DPP4 inhibition primarily targets ongoing fibrogenic processes rather than mitigating early inflammatory responses. We acknowledge that DPP4 inhibition may also modulate intestinal inflammation, as reported in previous studies;^40‐42^ however, our experimental design specifically focused on evaluating its direct antifibrotic effects during the active fibrogenic phase. Furthermore, as both inhibitors converge on the PI3K-AKT-mediated fibrotic signalling in myofibroblasts, their efficacy is unlikely to result solely from anti-inflammatory actions. These findings are consistent with our *in vitro* observations and position dual DPP4 inhibition as a conceptually novel strategy for CD-associated fibrosis, simultaneously targeting host stromal activation and microbiota-driven fibrogenic cues. While these results demonstrate the benefit of dual DPP4 inhibition, the relative contributions of membranous, soluble, and microbial DPP4 to fibrosis progression remain to be fully defined. Our data support a model of synergy rather than primacy. Both sources are concomitantly upregulated in fibrosis, and inhibition of either source alone confers partial protection, whereas dual inhibition yields superior efficacy *in vivo*, suggesting they form a reinforced “DPP4 pool” and act in a complementary rather than redundant manner. Critically, equivalent concentrations of host and microbial DPP4 exhibit comparable potency in activating human intestinal myofibroblasts via the shared PI3K-AKT pathway. Thus, their relative impact *in vivo* likely hinges on local abundance and duration of exposure rather than intrinsic efficacy. We propose that host DPP4 acts as a persistent endogenous driver, while microbiota-derived DPP4 serves as a luminal amplifier. Future studies using DPP4 knockout mice (to exclude both membrane-bound and soluble DPP4) and germ-free mice (to exclude bacteria-derived DPP4) will be required to clarify their precise dynamic contributions. The term “dual inhibition” is used herein to describe the specific experimental design and observed therapeutic effects, without implying that comprehensive blockade of all DPP4 sources is strictly necessary. Additionally, although this design does not fully represent a fibrosis reversal model, it approximates a clinically relevant late-phase intervention scenario. Future studies using delayed treatment protocols or fibrosis-specific reporters will be essential to evaluate the potential of DPP4 inhibitors in reversing established intestinal fibrosis.

## Conclusion

In conclusion, our study unveils a multifactorial model of intestinal fibrosis in CD, wherein both host-derived (membrane-bound and soluble) and microbiota-secreted DPP4 converge to drive myofibroblast activation, ECM remodelling, and stricture formation. These findings not only redefine the role of DPP4 beyond glycemic regulation, but also establish a framework for microbiota-targeted antifibrotic therapies in CD. Future studies should investigate pan-DPP4 inhibition strategies and validate their therapeutic efficacy using patient-derived organoid models and clinical trials.

## Supplementary Material

Supplementary material Supplementary material

## Data Availability

The metagenomic sequencing data generated in this study have been deposited in the National Genomics Data Centre (NGDC, https://ngdc.cncb.ac.cn/) under BioProject accession number PRJCA045251.
