# Supplementary material for: Dual-source DPP4 drives intestinal fibrosis in Crohn’s disease: synergistic therapeutic targeting of host and microbiota pathways
Source: Gut Microbes. 2025 Dec 3;17(1):2593119. doi: 10.1080/19490976.2025.2593119 (PMC12688270; doi:10.1080/19490976.2025.2593119)
Supplement: Supplementary material [file KGMI_A_2593119_SM5713.docx]

**Supplementary materials**

**Supplementary Table 1**

1. **The clinical characteristics of subjects. (Figure 1D-H)**

|  | **Control** | **CD** |
| --- | --- | --- |
| **Number** | 5 | 12 |
| **Age (years)** | 38.6 ± 13.3 | 35.7 ± 12.3 |
| **Gender** |  |  |
| male | 3 | 6 |
| female | 2 | 6 |
| **Disease duration (months)** |  | 43.3 ± 30.4 |
| **Current therapy** |  |  |
| 5-aminosalicylates |  | 9 |
| Immunosuppressants |  | 5 |
| Biologics |  | 7 |
| Nutritional therapy |  | 7 |
| **Disease location (CD)^*^** |  |  |
| L1 |  | 5 |
| L2 |  | 2 |
| L3 |  | 5 |
| L4 |  | 5 |

1. **The clinical characteristics of subjects. (Figure 4A)**

|  | **Control** | **CD without stenosis** | **CD with stenosis** |
| --- | --- | --- | --- |
| **Number** | 10 | 20 | 20 |
| **Age (years)** | 35.0 ± 10.70 | 33.8 ± 14.1 | 36.5 ± 13.3 |
| **Gender** |  |  |  |
| Male | 5 | 9 | 12 |
| Female | 5 | 11 | 8 |
| **Disease duration (months)** |  | 51.7± 30.4 | 50.0 ± 43.1 |
| **Current therapy** |  |  |  |
| 5-aminosalicylates |  | 6 | 6 |
| Immunosuppressants |  | 4 | 6 |
| Biologics |  | 15 | 12 |
| Nutritional therapy |  | 2 | 11 |
| **Disease location (CD)^*^** |  |  |  |
| L1 |  | 6 | 3 |
| L2 |  | 3 | 5 |
| L3 |  | 11 | 12 |
| L4 |  | 6 | 9 |

1. **The clinical characteristics of subjects. (Figure 6B-E, S3A-C)**

|  | **Control** | **CD without stenosis** | **CD with stenosis** |
| --- | --- | --- | --- |
| **Number** | 10 | 20 | 20 |
| **Age (years)** | 41.1 ± 9.10 | 38.2 ± 11.5 | 37.6 ± 13.6 |
| **Gender** |  |  |  |
| Male | 4 | 11 | 12 |
| Female | 6 | 9 | 8 |
| **Disease duration (months)** |  | 52.3 ± 29.3 | 53.3 ± 32.9 |
| **Current therapy** |  |  |  |
| 5-aminosalicylates |  | 6 | 8 |
| Immunosuppressants |  | 6 | 6 |
| Biologics |  | 10 | 8 |
| Nutritional therapy |  | 4 | 10 |
| **Disease location (CD)^*^** |  |  |  |
| L1 |  | 6 | 8 |
| L2 |  | 2 | 3 |
| L3 |  | 12 | 9 |
| L4 |  | 6 | 6 |

**Supplementary Table 2. The amino acid sequence of btDPP4**

| **Protein** | **Sequences** |
| --- | --- |
| btDPP4 | MHHHHHHQKALDLKDITSGRFRPENIQGVIPMPDGEHYTQMSADGTQIIKYSFRTGEKVEVIFDVNQARECDFKNFDSYQFSPDGDKLLIATRTTPIYRHSYTAVHYIYPLKRNDKGVTTNNIIERLSDGGPQQVPVFSPDGTMIAFVRDNNIFLVKLLYGNSESQVTEDGKQNSVLNGIPDWVYEEEFGFNRALEFSADNTMIAFIRFDESEVPSYSFPMFAGEAPQITPLKDYPGEYTYKYPKAGYPNSKVEVRTYDIKSHVTRTMKLPIDADGYIPRIRFTKDASKLAVMTLNRHQDRFDLYFADPRSTLCKLVLRDESPYYIKENVFDNIKFYPETFSLLSERDGFSHLYWYSMGGNLIKKVTNGKYEVKDFLGYDEADGSFYYTSNEESPLRKAVYKIDKKGKKLKLSQREGTNTPLFSQSMKYYMNKFSNLDTPMLVTLNDNTGKTLKTLINNDQLKQTLSGYAIPQKEFFTFQTTDGVTLNGWMMKPANFSTSKKYPVLMYQYSGPGSQQVLDTWGISWETYMASLGYIVVCVDGRGTGGRGEAFEKCTYLKIGVKEAKDQVETALYLGKQPYVDKDRIGIWGWSYGGYMTLMSMSEGTPVFKAGVAVAAPTDWRFYDTIYTERFMRTPKENAEGYKESSAFTRADKLHGNLLLVHGMADDNVHFQNCAEYAEHLVQLGKQFDMQVYTNRNHGIYGGNTRQHLYTRLTNFFLNNL |

**Supplementary Table 3. Sequences of primers for RT-qPCR**

| Speices | Gene | Direction | Primer sequence (5’ to 3’) |
| --- | --- | --- | --- |
| Human | *DPP4* | Forward | AGTGGCACGGCAACACATT |
|  |  | Reverse | AGAGCTTCTATCCCGATGACTT |
| Human | *COL1A1* | Forward | GAGGGCCAAGACGAAGACATC |
|  |  | Reverse | CAGATCACGTCATCGCACAAC |
| Human | *ACTA2* | Forward | AAAAGACAGCTACGTGGGTGA |
|  |  | Reverse | GCCATGTTCTATCGGGTACTTC |
| Human | GAPDH | Forward | CACCATCTTCCAGGAGCGAG |
|  |  | Reverse | GATGGCATGGACTGTGGTCA |
| Mouse | *Col6a1* | Forward | CTGCTGCTACAAGCCTGCT |
|  |  | Reverse | GCACGAAGAATAGATCCACAGGG |
| Mouse | *Col1a1* | Forward | TAAGGGTCCCCAATGGTGAGA |
|  |  | Reverse | GGGTCCCTCGACTCCTACAT |
| Mouse | *Gapdh* | Forward | AGGTCGGTGTGAACGGATTTG |
|  |  | Reverse | TGTAGACCATGTAGTTGAGGTCA |
| Bacteria | *bdDpp4* | Forward | TCGTCGTTCGTTTACTGCCA |
|  |  | Reverse | CTGTTGTGGGCCGTTTTCTG |
| Bacteria | *bfDpp4* | Forward | ATTGCAACCGAAACCAAGCC |
|  |  | Reverse | GACAAACGCCACCAGGTTTC |
| Bacteria | *btDpp4* | Forward | AGCCGTCTGTTGCGTCATAA |
|  |  | Reverse | TAGCGAACGTGACGGTTTCA |
| Bacteria | *bvDpp4* | Forward | CGGTCCACAACAGGTTCCTT |
|  |  | Reverse | ACGGTTGAAGCCGAATTCCT |
| Bacteria | 16S rRNA | Forward | GTGCCAGCMGCCGCGGTAA |
|  |  | Reverse | GACTACCAGGGTATCTAATCC |
| Bacteria | *Bt*-16S | Forward | TGCCGTCGTAAGATGTGAGG |
|  |  | Reverse | ATCCAGCTTGACGAAGTCGG |
| Bacteria | *E. coli*-16S | Forward | GCAACGGCGTTCGCTTGAA |
|  |  | Reverse | TTGCCGAATGTTCTCTGGCA |
| Bacteria | attλ his | Forward | CACCACCACCACCACCACTAAATACTAACTTGAGCGAAAC |
|  | pelB-DPP4 | Reverse | CAAGTGCCTTTTGTCCTTGcatGGCCATCGCCGGCTGGGC |
| Bacteria | DPP4-pelB | Forward | CCCAGCCGGCGATGGCCatgCAAGGACAAAAGGCACTTGA |
|  | DPP4-attλ | Reverse | TAGTGGTGGTGGTGGTGGTGCAAATTGTTCAGGAAGAAGT |
| Bacteria | attλ-up123 | Forward | GTTGAGCTACAGGCGGTCAGCGT |
|  | attλ-down123 | Reverse | GCCTCGATTACTGCGATGTTTAG |

**Figure S1. Identification of fibrosis-associated gene modules by WGCNA and protein–protein interaction analysis.**


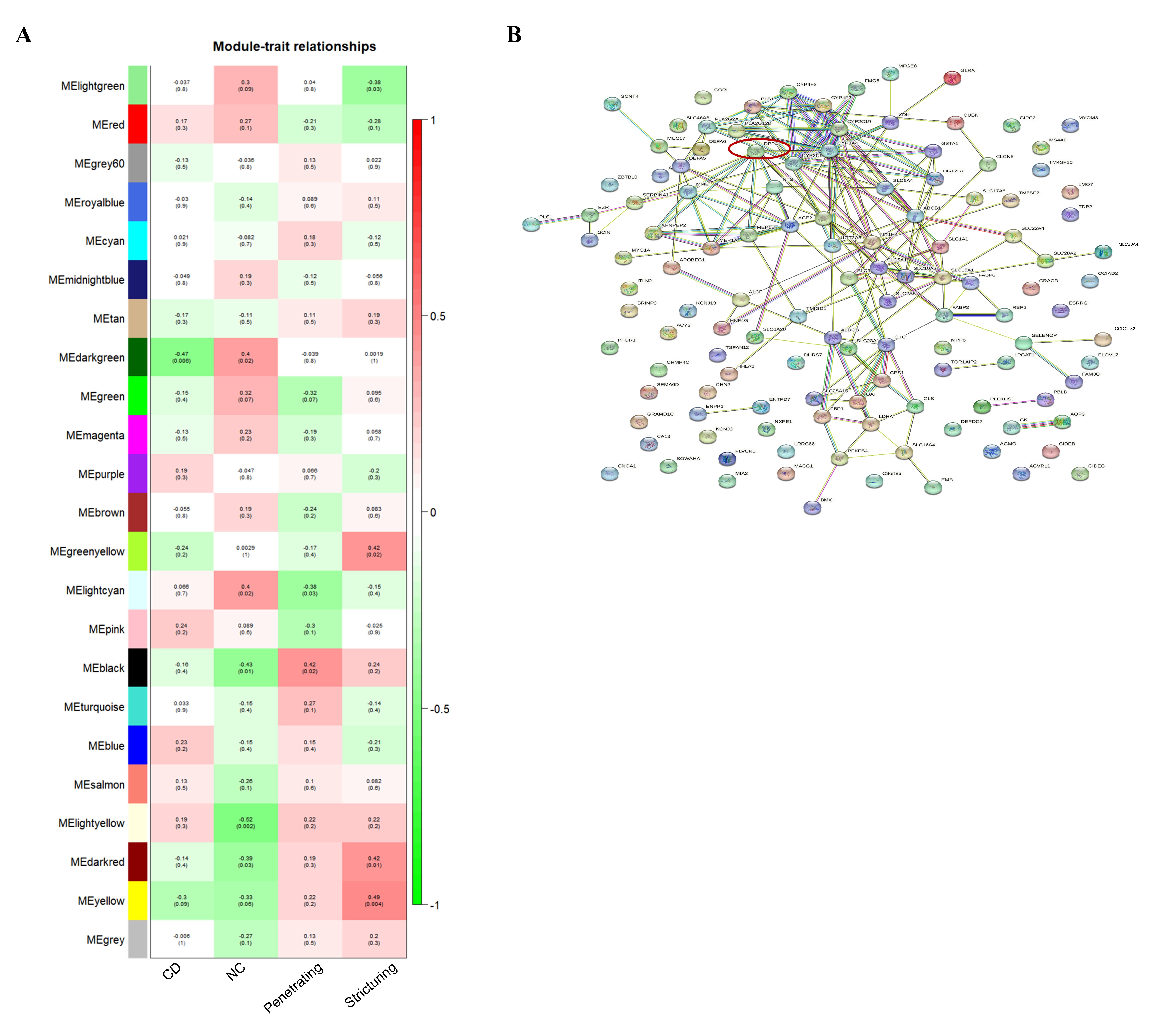


**(A)** WGCNA weighted correlation network was used to analyze the relationship between genes and phenotypes and to cluster modules. **(B)** The intersection of ME yellow module gene and differential gene was analyzed in the STRING database for protein interaction.

**Figure S2. Flow cytometric detection of surface DPP4 expression in primary HIMFs.**


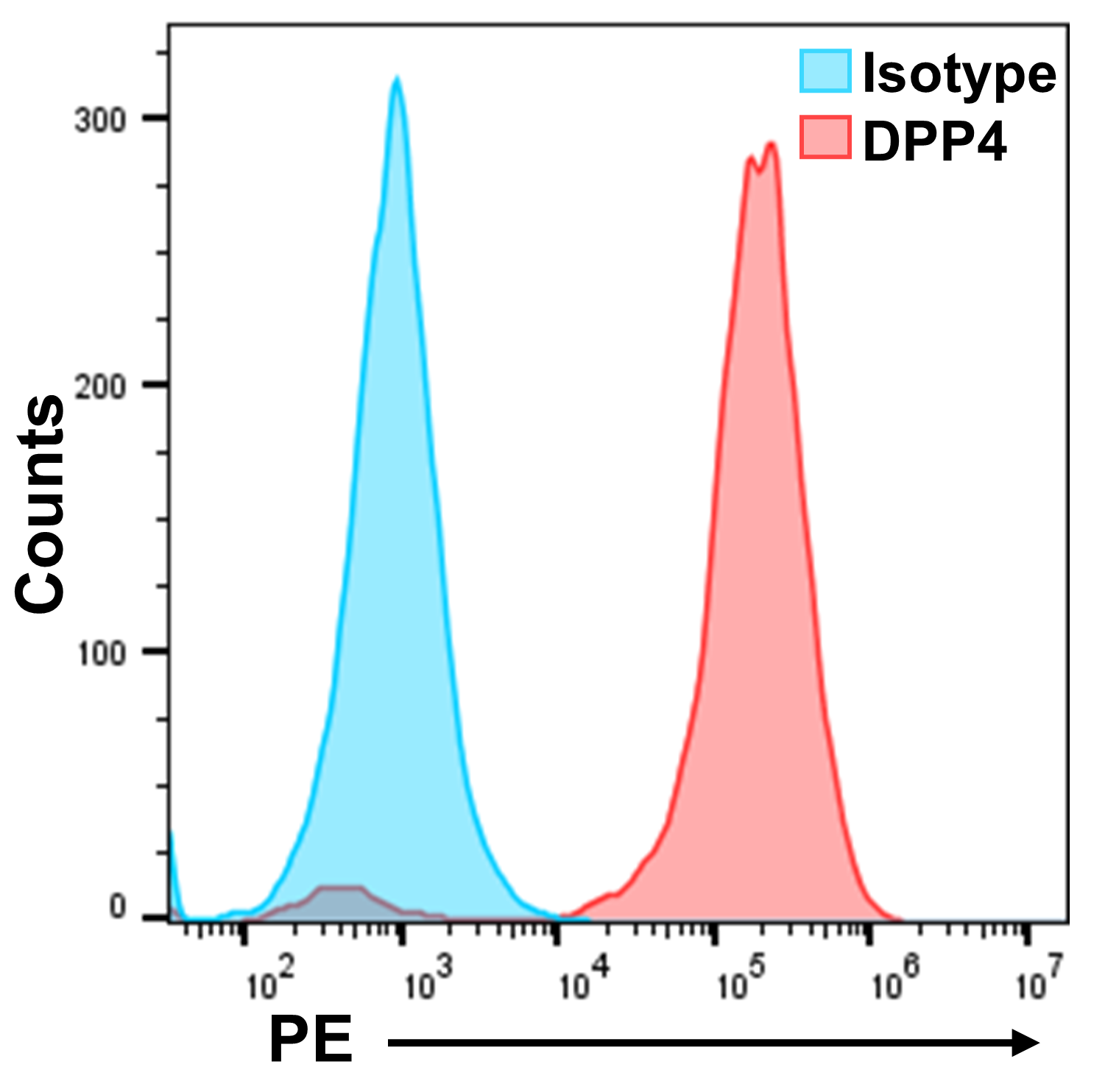


Representative histogram showing a distinct DPP4⁺ population compared to isotype control.

**Figure S3. Pathway enrichment analysis of DPP4⁺ versus DPP4⁻ fibroblasts from stenotic CD tissues.**

**
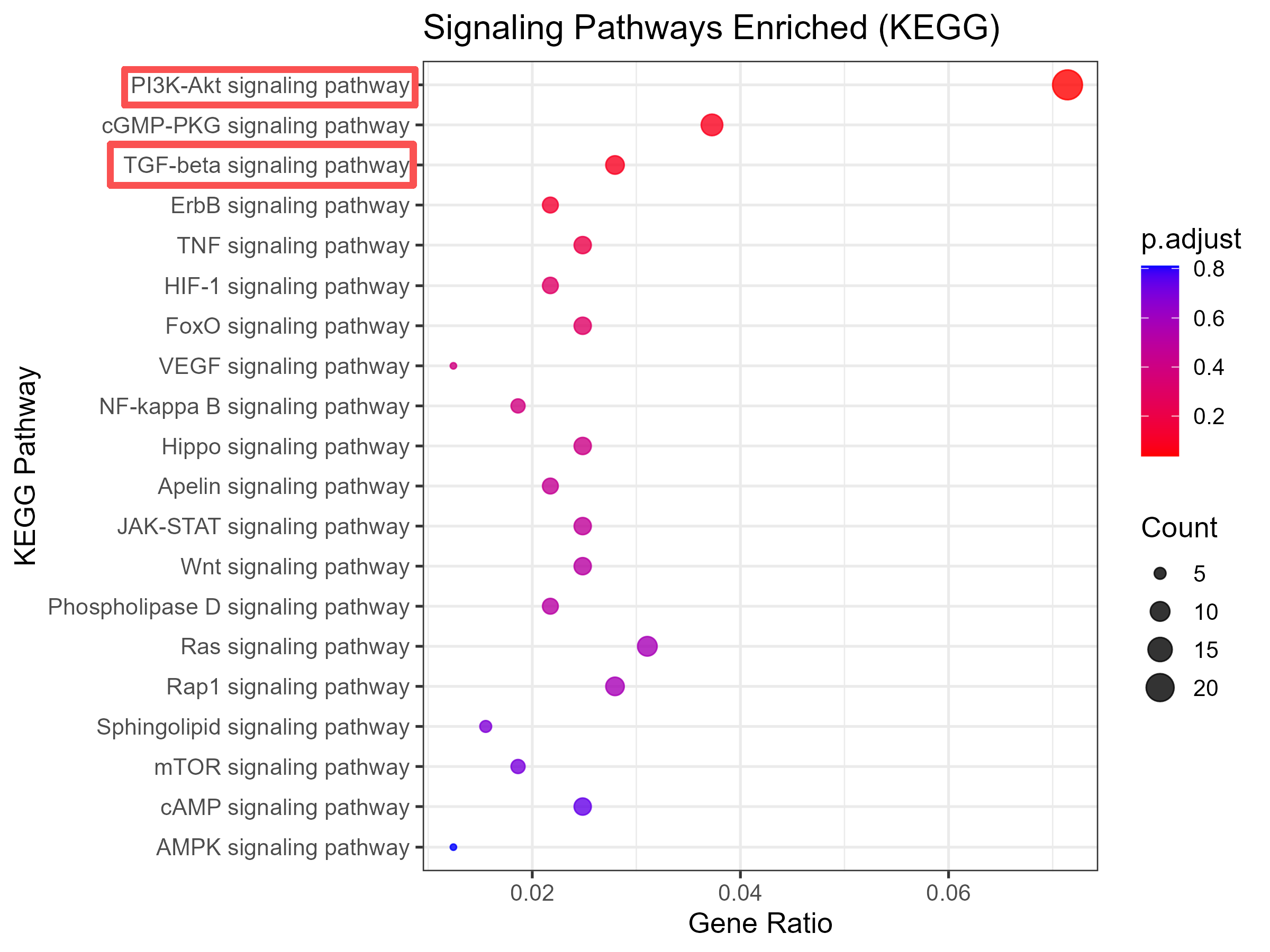
**

Differentially expressed genes (DEGs) between DPP4⁺ and DPP4⁻ fibroblasts were identified from a single-cell RNA-seq dataset of full-thickness stricturing CD intestinal tissues. KEGG pathway enrichment analysis was performed using the clusterProfiler package in R to characterize signaling pathways associated with DPP4⁺ fibroblasts. The bubble plot displays KEGG pathways significantly enriched in DPP4⁺ fibroblasts, with point size representing the number of genes and color representing the adjusted *p*-value.

**Figure S4. Taxonomic assignment, expression validation, and secretion profile of microbiota-derived DPP4 in CD.**

**

**

**(A)** Genus-level contribution of microbial *Dpp4* genes in CD patients with stenosis, highlighting the top five contributing genera ranked by relative abundance. **(B)** Relative abundance of microbial *Dpp4* genes from different *Bacteroides* species, as determined from fecal metagenomic data across all samples. **(C)** RT-qPCR quantification of microbial *Dpp4* mRNA in feces from controls, CD patients with stenosis and those without. **(D)** Fecal microbial *Dpp4* mRNA expression in DSS-induced chronic colitis mice versus controls. **(E)** Circular genome visualization of *B. thetaiotaomicron* was performed using CGView Server based on the GenBank assembly GCA_014131755.1. The DPP4-encoding locus was annotated according to the reference annotation file. **(F)** Growth curve of *B. thetaiotaomicron* cultured under anaerobic conditions, showing OD600 measurements over time. **(G)** Quantification of DPP4 enzymatic activity in culture supernatants of *B. thetaiotaomicron* collected at 8 h, 16 h, and 24 h. Activity was measured using the Gly-Pro-pNA substrate. BHI medium alone was used as a negative control.

**Figure S5. Validation of engineered *E. coli* overexpressing btDPP4.**

**
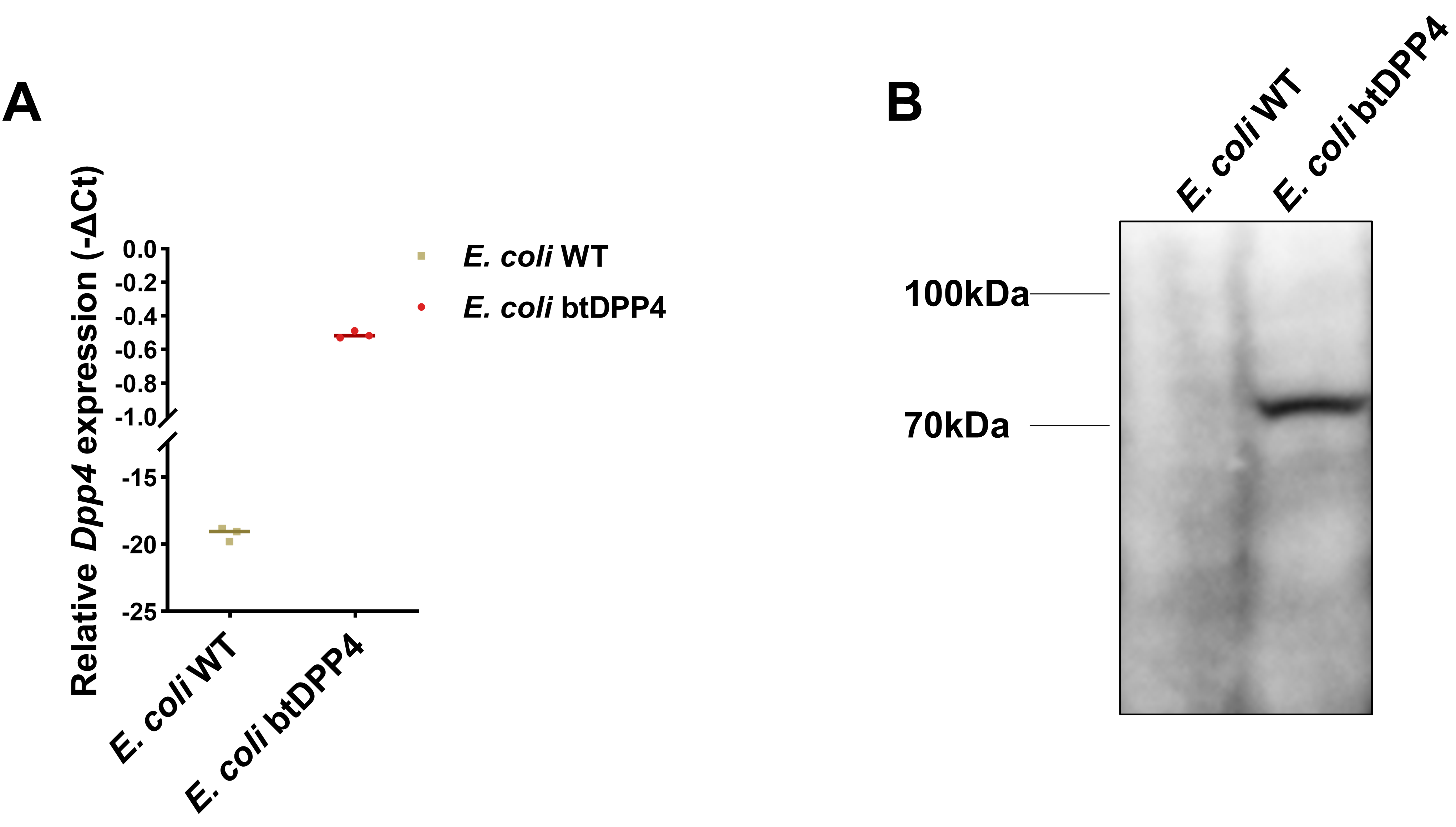
**

**(A)** Quantitative PCR analysis of *Dpp4* expression in wild-type *E. coli* (*E. coli* WT) and engineered strains overexpressing btDPP4 (*E. coli* btDPP4). **(B)** Western blot analysis of recombinant His-tagged btDPP4 protein expression in engineered *E. coli* strains compared with WT controls.
